# Validation of ketamine as a pharmacological model of thalamic dysconnectivity across the illness course of schizophrenia

**DOI:** 10.1038/s41380-022-01502-0

**Published:** 2022-04-14

**Authors:** Samantha V. Abram, Brian J. Roach, Susanna L. Fryer, Vince D. Calhoun, Adrian Preda, Theo G. M. van Erp, Juan R. Bustillo, Kelvin O. Lim, Rachel L. Loewy, Barbara K. Stuart, John H. Krystal, Judith M. Ford, Daniel H. Mathalon

**Affiliations:** 1 Sierra Pacific Mental Illness Research Education and Clinical Centers, San Francisco Veterans Affairs Medical Center, and the University of California, San Francisco, CA USA; 2 San Francisco Veterans Affairs Medical Center, 4150 Clement Street, San Francisco, CA 94121 USA; 3 Department of Psychiatry and Behavioral Sciences, University of California San Francisco, 505 Parnassus Avenue, San Francisco, CA 94143 USA; 4 Tri-institutional Center for Translational Research in Neuroimaging and Data Science (TReNDS), Georgia State, Georgia Tech, Emory, Atlanta, GA 30332 USA; 5 Department of Psychiatry and Human Behavior, University of California Irvine Medical Center, 101 The City Dr. S, Orange, CA 92868 USA; 6 Clinical Translational Neuroscience Laboratory, Department of Psychiatry and Human Behavior, University of California Irvine, 5251 California Ave, Irvine, CA 92617 USA; 7 Center for the Neurobiology of Learning and Memory, University of California Irvine, 309 Qureshey Research Lab, Irvine, CA 92697 USA; 8 Department of Psychiatry and Behavioral Sciences, University of New Mexico Health Sciences Center, Albuquerque, NM 87111 USA; 9 Department of Psychiatry, University of Minnesota, Minneapolis, MN 55454 USA; 10 Department of Psychiatry, Yale University School of Medicine, New Haven, CT 06510 USA

**Keywords:** Neuroscience, Psychology

## Abstract

*N*-methyl-D-aspartate receptor (NMDAR) hypofunction is a leading pathophysiological model of schizophrenia. Resting-state functional magnetic resonance imaging (rsfMRI) studies demonstrate a thalamic dysconnectivity pattern in schizophrenia involving excessive connectivity with sensory regions and deficient connectivity with frontal, cerebellar, and thalamic regions. The NMDAR antagonist ketamine, when administered at sub-anesthetic doses to healthy volunteers, induces transient schizophrenia-like symptoms and alters rsfMRI thalamic connectivity. However, the extent to which ketamine-induced thalamic dysconnectivity resembles schizophrenia thalamic dysconnectivity has not been directly tested. The current double-blind, placebo-controlled study derived an NMDAR hypofunction model of thalamic dysconnectivity from healthy volunteers undergoing ketamine infusions during rsfMRI. To assess whether ketamine-induced thalamic dysconnectivity was mediated by excess glutamate release, we tested whether pre-treatment with lamotrigine, a glutamate release inhibitor, attenuated ketamine’s effects. Ketamine produced robust thalamo-cortical hyper-connectivity with sensory and motor regions that was not reduced by lamotrigine pre-treatment. To test whether the ketamine thalamic dysconnectivity pattern resembled the schizophrenia pattern, a whole-brain template representing ketamine’s thalamic dysconnectivity effect was correlated with individual participant rsfMRI thalamic dysconnectivity maps, generating “ketamine similarity coefficients” for people with chronic (SZ) and early illness (ESZ) schizophrenia, individuals at clinical high-risk for psychosis (CHR-P), and healthy controls (HC). Similarity coefficients were higher in SZ and ESZ than in HC, with CHR-P showing an intermediate trend. Higher ketamine similarity coefficients correlated with greater hallucination severity in SZ. Thus, NMDAR hypofunction, modeled with ketamine, reproduces the thalamic hyper-connectivity observed in schizophrenia across its illness course, including the CHR-P period preceding psychosis onset, and may contribute to hallucination severity.

## Introduction

The thalamus plays a fundamental role in how the brain coordinates information to support sensory processing and cognition [1, 2]. Breakdowns in thalamic coordination are highly replicated in schizophrenia (SZ) [3–8] and implicated in an array of clinical and cognitive symptoms [9, 10]. Deficits in *N*-methyl-D-aspartate receptor (NMDAR) signaling could underlie disturbed thalamic circuitry in SZ [11]. Ketamine is a noncompetitive NMDAR antagonist that produces transient SZ-like symptoms [12, 13] and disrupts thalamic connectivity when administered to healthy volunteers [14–17]. However, the extent to which the aberrant connectivity pattern produced by ketamine resembles the SZ pattern has not been directly tested. Here, we derive an NMDAR hypofunction model of thalamic dysconnectivity from healthy volunteers administered ketamine during resting-state functional magnetic resonance imaging (rsfMRI), and directly test whether this model resembles the dysconnectivity pattern observed in people with SZ.

It has become increasingly apparent that no single brain region can account for the range of symptoms and functional impairments observed in SZ; rather, coordination abnormalities across brain regions likely give rise to diverse cognitive and behavioral impairments [18, 19]. The thalamus is key to understanding these systemic breakdowns [20], as it is a richly interconnected hub for many functional brain networks [21, 22]. Several rsfMRI studies show that, when compared to healthy controls (HC), people with SZ exhibit thalamic hyper-connectivity with sensory regions (e.g., auditory, visual, motor, and association cortices) and hypo-connectivity with prefrontal, cerebellar, and thalamic regions [5, 6, 23, 24]. The term “dysconnectivity” was adopted to refer to the concurrent hyper- and hypo-connectivity patterns characteristic of SZ. A similar pattern is seen in individuals at clinical high-risk for psychosis (CHR-P) [25–27], particularly among those who convert to full psychosis [26]. In a cross-sectional study, we found that at-risk individuals showed an intermediate level of thalamic dysconnectivity, raising the possibility that thalamic dysconnectivity begins before psychosis onset, and worsens with illness course [27].

Previously, we found thalamic dysconnectivity in SZ to be associated with more severe positive symptoms [23]. However, a more recent meta-analytic review of thalamic connectivity studies in individuals with psychotic disorders indicates that clinical correlations are evident for positive, negative, and general symptoms [7], although the correlations tended to be small, variable, and not specific to a particular symptom domain. These variable findings could result from the use of coarse, subjective clinical rating scales that do not map directly onto specific brain circuits [8]. Targeting more specific clinical phenomena with large samples may help elucidate the consequences of thalamic dysconnectivity (as done by [23]).

The NMDAR hypofunction model [28–30] posits that glutamate synaptic signaling abnormalities associated with SZ are mimicked by the acute and chronic effects of NMDAR antagonists [31]. In SZ, deficient glutamate NMDAR signaling across development contributes to the emergence of deficits in GABA release and signaling. This paradoxically leads to states where there are both deficits in glutamate NMDAR synaptic signaling and disinhibition of glutamate release [32]. As SZ progresses, this picture is further complicated by excessive synaptic pruning [33, 34]. The acute administration of the NMDAR antagonist, ketamine, yields both deficits in glutamate NMDAR synaptic signaling and inhibition of GABAergic interneurons [35], resulting in downstream hyperglutamatergia and associated network disinhibition. Chronic ketamine administration reduces the integrity of GABA neuronal populations and synaptic loss [36]. Thus, acute ketamine mimics the pathophysiological processes present in the early stages of SZ [3, 14], while chronic NMDAR blockade with ketamine mimics the pathophysiological changes that arise later in the illness [28].

A cornerstone of the NMDAR hypofunction model of SZ is the observation that NMDAR antagonists, including ketamine, induce transient, positive, negative, and cognitive symptoms in healthy volunteers that resemble symptoms of SZ [12, 37]. Psychotomimetic effects of NMDAR antagonists may be at least partially mediated through the thalamus [38], as injection of an NMDAR antagonist into the rodent thalamus led to altered neural oscillations in the prefrontal cortex [39] (similar to SZ [40]), and reduced 40-Hz auditory steady-state responses [41] (consistent with SZ deficits [42]). Two recent reports found that ketamine administration led to increased thalamic and sensory cortex connectivity in humans [14, 16], further implicating NMDAR signaling in thalamic dysfunction. However, these studies did not directly test whether thalamic dysconnectivity induced by NMDAR antagonists resembled thalamic dysconnectivity observed in SZ, and whether the degree to which people with SZ show the NMDAR antagonist dysconnectivity pattern was associated with clinical symptom severity.

The current study derives a ketamine model of thalamic dysconnectivity to test whether NMDAR hypofunction can account for the pattern of thalamic dysconnectivity observed in SZ. Using a seed-based approach, we examine thalamic connectivity changes following ketamine administration to healthy volunteers. We then assess whether ketamine’s effects are mediated by downstream increases in glutamate release, rather than its proximal blockade of glutamate transmission at NMDARs. Specifically, we test whether pre-treatment with lamotrigine, a glutamate release inhibitor [43], attenuates ketamine’s effects on thalamic connectivity. Finally, we evaluate the extent to which the ketamine-induced thalamic dysconnectivity pattern resembles the SZ pattern and accounts for variation in specific clinical symptoms. To this end, we derive a template brain map of ketamine’s thalamic dysconnectivity effects and correlate it with individual participant rsfMRI thalamic connectivity maps from a multi-site sample of individuals with chronic SZ and HC, and a separate sample of individuals early in the schizophrenia illness course (ESZ), individuals at CHR-P, and HC. We hypothesize that the ketamine thalamic dysconnectivity template will show stronger positive correlations with thalamic dysconnectivity maps in chronic SZ relative to HC, and in ESZ relative to HC, with CHR-P falling intermediately between them. We also hypothesize that greater similarity to the ketamine-induced thalamic dysconnectivity pattern will correlate with greater symptom severity.

## Methods

### Ketamine – lamotrigine study

#### Participants

Healthy men (*N* = 18; mean age = 27.96 ± 3.97 years) were recruited in New Haven, Connecticut. The targeted sample size was based on evidence that a comparable sample size yielded detectable behavioral effects associated with lamotrigine pre-treatment prior to ketamine challenge [44]. Participants were excluded for an unstable medical illness, psychiatric disorder, first-degree relative with a psychotic disorder, elevated score on a Psychosis Proneness Scale [45], or MRI contraindication. Study procedures were approved by the Institutional Review Boards at Yale University School of Medicine and the US Department of Veterans Affairs, West Haven. All participants provided written informed consent.

#### Drug procedure and randomization

Using a double-blind, placebo-controlled, crossover design, participants completed three test days in a randomized order (Supplementary Fig. S1). Participants and investigators were blinded to the test day order. The test days were as follows: Placebo Lamotrigine – Placebo Ketamine: oral placebo lamotrigine 1.5 h before a first (single-blind) intravenous placebo ketamine (saline) infusion, followed 49 min later by a second (double-blind) intravenous placebo ketamine (saline) infusion; Placebo Lamotrigine – Active Ketamine: oral placebo lamotrigine 1.5 h before a first (single-blind) intravenous placebo ketamine (saline) infusion, followed 49 min later by a second (double-blind) intravenous active ketamine infusion; and Active Lamotrigine – Active Ketamine: oral active lamotrigine 1.5 h prior to a first (single-blind) intravenous placebo ketamine (saline) infusion, followed 49 min later by a second (double-blind) intravenous active ketamine infusion. Study sessions occurred at least one week apart. Supplementary Table S1 contains the study procedure timeline.

Similar to other reports [46, 47], ketamine was administered as a 2-min bolus (0.23 mg/kg) followed by 0.58 mg/kg/h steady infusion over about 70 min. The ketamine dose was comparable to doses from studies showing altered prefrontal function during infusion in healthy individuals [46, 48], and the lamotrigine dose (300 mg) was chosen based on safety considerations and evidence showing that this lamotrigine dose attenuated ketamine’s behavioral effects [44]. Additional dosing details are in Supplementary Materials. Ketamine produced substantial increases in psychotomimetic effects, and pre-treatment with lamotrigine did not attenuate those increases (Supplementary Materials).

#### Neuroimaging acquisition and data processing

Data were collected on a 3T SIEMENS TRIO scanner. Functional data included six 7-min runs of a visual oddball task (voxel size = 3.4 × 3.4 × 4.5 mm, repetition time = 2 s, total volumes = 210) collected for each test day: three runs during the initial saline infusion, and three during the second active ketamine (or placebo ketamine) saline infusion. Functional connectivity measures were computed using the oddball task runs after regressing out task-related brain activity (a validated approach implemented previously [49, 50]), as continuous rsfMRI data were not collected for this study (see Supplementary Materials for oddball task description). A high-resolution T1-weighted structural image (MPRAGE) was collected for co-registration with functional data. Pre-processing and denoising steps are detailed in Supplementary Materials.

First-level seed-based connectivity analyses were carried out using FEAT in FSL version 6.0.0 (https://fsl.fmrib.ox.ac.uk/fsl/fslwiki/FEAT/). A participant’s fMRI scan time-series was regressed on the thalamus seed’s time-series (extracted from pre-smoothed images), and a set of nuisance regressors that are described in Supplementary Materials. We used a whole thalamus seed to compare ketamine’s effects with prior SZ connectivity studies that used a whole thalamus seed, including our own [23, 27]. We defined the bilateral thalamus seed using FSL’s Harvard-Oxford Subcortical Structural Atlas probabilistic maps. To ensure our seed represented thalamus activity rather than extra-thalamic activity, we used a stringent 80% probability threshold. First-level models produced whole-brain voxel-wise beta maps, separately for each participant, drug condition, and run (three runs per drug condition); the resulting maps reflected the correlation between the thalamus seed’s time-series and the time-series of every voxel in the brain.

Second-level fixed-effects models produced within-participant, within-day contrast maps for the Placebo Lamotrigine – Placebo Ketamine and Placebo Lamotrigine – Active Ketamine test days. For the Placebo Lamotrigine – Placebo Ketamine day, this yielded a within-participant contrast map reflecting placebo ketamine (runs 4–6) versus saline (runs 1–3). For the Placebo Lamotrigine – Active Ketamine day, this yielded a within-participant contrast map reflecting active ketamine (runs 4–6) versus saline (runs 1–3). We then calculated between-day contrast maps for each participant that compared these two within-day contrasts (i.e., [active ketamine – saline] versus [placebo ketamine – saline]); this final map isolated the effect of active ketamine versus placebo ketamine, accounting for run order effects. Runs 2–5 from one participant from the Placebo Lamotrigine – Placebo Ketamine day were removed due to scanner-induced artifacts; their corresponding contrast was thus calculated as placebo ketamine (run 6) versus saline (run 1). The group-level results were comparable with or without this participant.

#### Group-level modeling of ketamine effects on thalamic dysconnectivity

Final group-level random-effects one-sample *t*-test analyses were run separately on placebo ketamine and active ketamine connectivity maps to identify clusters showing significant positive or negative thalamic connectivity. In addition, one-sample *t*-tests were run on the [active ketamine – saline] versus [placebo ketamine – saline] contrast map. Group-level *t*-maps were transformed into *z*-maps and warped into MNI standard space. We implemented a voxel-wise cluster defining threshold (*z* > 3.29, *p* < 0.001), and a corrected cluster significance threshold (*p* < 0.05, two-tailed). For each significant cluster from the contrast analyses, mean connectivity values were extracted for each participant and drug condition, allowing the distribution under each condition to be plotted and effect sizes to be computed.

We also probed whether ketamine-induced thalamic dysconnectivity correlated with ketamine-induced psychotomimetic effects in healthy volunteers; these results were not significant (Supplementary Materials).

#### Group-level modeling of lamotrigine pre-treatment effects on ketamine-induced thalamic dysconnectivity

Thalamic connectivity maps were derived for the Active Lamotrigine – Active Ketamine test day using the same procedures described above. First-level models produced whole-brain voxel-wise beta maps for each Active Lamotrigine – Active Ketamine run. Second-level fixed-effects models averaged runs 4–6 to produce a single [active lamotrigine + active ketamine] map per participant. Significant regional clusters from the [active ketamine – saline] versus [placebo ketamine – saline] contrast were saved as region of interest (ROI) masks. Mean thalamic connectivity values were extracted for each ROI cluster and drug condition. Repeated measures ANOVAs were used to compare average thalamic connectivity in these ROIs across the three conditions (i.e., placebo ketamine, active ketamine, active lamotrigine + active ketamine); follow-up pairwise tests based on each ANOVA assessed whether lamotrigine pre-treatment altered or attenuated ketamine’s dysconnectivity effects in these ROIs, and whether ketamine with lamotrigine pre-treatment still produced thalamic dysconnectivity relative to placebo ketamine in these ROIs. We adjusted for multiple comparisons across ROI models using Benjamini and Hochberg’s false discovery rate (FDR) algorithm [51]. Comparison of thalamic connectivity during runs 1–3 across test days are reported in Supplementary Materials.

### Schizophrenia versus healthy control study

#### Participants

SZ (*n* = 183; mean age = 38.73 ± 11.53 years, 75% male) and HC (*n* = 178; mean age = 37.70 ± 11.19 years, 71% male) were recruited from seven sites as part of the Function Biomedical Informatics Research Network (FBIRN) study. Enrollment and exclusion criteria for this sample are described in Supplementary Materials and elsewhere [23].

#### Neuroimaging data processing

Whole-brain rsfMRI connectivity maps were generated for each participant in SPM8 using a bilateral thalamus seed and expressed as Fisher *r*-to-*z* transformed correlation maps [23]. Data denoising procedures are found in Supplementary Materials. Using HC from each FBIRN site as normative data, each participant’s thalamic connectivity map was site-corrected; i.e., *z*-scores were calculated as the difference between a participant’s observed value and the value predicted for their site, divided by the standard error of the regression from the HC regression model. Resulting *z*-score maps expressed voxel values as deviations, in standard deviation units, from the values expected for a HC at the same study site.

#### Quantifying the similarity with ketamine-induced thalamic dysconnectivity

The unthresholded group-level [active ketamine – saline] > [placebo ketamine – saline] contrast map (i.e., “ketamine template”) and the individual SZ and HC connectivity maps were transformed from 3D (with *x*, *y*, and *z* directions) into 1D vectors. Next, the 1D vector of thalamic connectivity *z*-scores for each HC and SZ was correlated with the 1D vector of *z*-statistics from the ketamine template. This yielded a single correlation for each participant reflecting the similarity of their map with ketamine-induced thalamic dysconnectivity (i.e., “ketamine similarity coefficients”). Similarity coefficients were Fisher *r*-to-*z* transformed and group differences were tested using an ANOVA. Because the ketamine template was generated from an exclusively male sample, the model included sex and a group × sex interaction term. The main effect of the site was also included to control for study site variance. Follow-up analyses within each group tested whether the mean similarity coefficient value was significantly different from 0.

For reference, Supplementary Fig. S2 shows the overlap between group-level ketamine thalamic dysconnectivity (i.e., the [active ketamine – saline] > [placebo ketamine – saline] contrast map) and group-level SZ thalamic dysconnectivity (i.e., the SZ > HC contrast map) (Supplementary Materials).

#### Ketamine similarity coefficients versus clinical symptoms

Separate regression models were used to evaluate relationships between ketamine similarity coefficients and SZ symptom scores rated using the Scale for the Assessment of Positive Symptoms (SAPS [52]) and the Scale for the Assessment of Negative Symptoms (SANS [53]). Symptom scores comprised the sum of individual items plus the respective global rating. Positive symptoms included Hallucinations, Delusions, Thought Disorder, and Bizarre Behavior, and negative symptoms included Affective Flattening, Alogia, Avolition/Apathy, and Anhedonia/Asociality. For each symptom score, only SZ with at least a mild global severity rating (≥2) were included [54, 55] (Supplementary Table S2 contains participant counts and distribution details). Symptom scores with skewed distributions were square-root transformed. Symptom scores were regressed on ketamine similarity coefficients, sex, and study site. Each model initially included the similarity coefficient × sex interaction to test for sex differences in the regression line slopes. Significant interactions were followed up with separate regression models in males and females. Non-significant interactions were dropped from the model, allowing a test of the common slope estimated across males and females. We FDR-adjusted across the eight tests.

#### Medication confound analyses

We computed chlorpromazine equivalents (CPZeq) for SZ (mean = 488.92 ± 1151.01 mg) [56]. To test for possible medication confounds, we correlated CPZeq values with ketamine similarity coefficients, finding a positive correlation (Spearman’s rho = 0.20, *p* = 0.02); here we used a non-parametric test given the non-normality of the CPZeq variable. Consequently, for any significant associations between symptoms and similarity coefficients, we tested whether those relationships remained significant in the subsample of SZ taking antipsychotic medication (*n* = 151), and then tested whether those effects remained when controlling for CPZeq.

### Early illness schizophrenia, clinical high-risk, versus healthy control study

#### Participants

ESZ (*n* = 74; mean age = 21.89 ± 4.22 years, 66% male; within 5 years of psychosis onset) and CHR-P (*n* = 45; mean age = 20.34 ± 4.71 years, 53% male) were recruited from early psychosis clinical programs at the University of California, San Francisco, and from community referrals. HC (*n* = 85; mean age = 22.65 ± 6.45 years, 60% male) were recruited from the local community. Enrollment and exclusion criteria for this sample are described in Supplementary Materials and elsewhere [27].

#### Neuroimaging data processing

After data denoising (using methods described in Supplementary Materials), whole-brain rsfMRI connectivity maps were generated for each participant in SPM8 using a bilateral thalamus seed and expressed as Fisher *r*-to-*z* transformed correlation maps [27]. Resulting thalamic connectivity maps were age-adjusted based on age-relationships modeled in the HC group, yielding individual *z*-score maps that expressed voxel values as deviations, in standard deviation units, from the values expected for a HC of the same age [27].

#### Quantifying the similarity with ketamine-induced thalamic dysconnectivity

Ketamine similarity coefficients were derived using the same procedures described above for the Schizophrenia versus Healthy Control Study. Resulting Fisher *r*-to-*z* similarity coefficients were compared across HC, CHR-P, and ESZ using a two-way group × sex ANOVA model. Follow-up analyses tested for pairwise group differences (FDR-adjusted), and whether the mean similarity coefficient was significantly different from 0 within each group.

#### Ketamine similarity coefficients versus clinical symptoms

For ESZ, we calculated regression models between ketamine similarity coefficients and the same symptom scores considered in the Schizophrenia versus Healthy Control Study, including ESZ with at least mild severity (≥2) on the global rating for that symptom (Supplementary Table S2 contains participant counts and distribution details). Symptom scores with skewed distributions were square-root transformed. Symptom scores were regressed on ketamine similarity coefficients and sex, testing initially for a similarity coefficient × sex interaction, and dropping the interaction term if not significant (to test the common slope). Following a significant interaction, we tested the slopes separately in males and females. We FDR-adjusted across the eight tests.

For CHR-P, we calculated regression models between ketamine similarity coefficients with Positive, Negative, Disorganized, and General symptoms measured using the Scale of Psychosis-risk Symptoms (SOPS [57]). We included sex and a similarity coefficient × sex term in each model, and FDR-adjusted across the four tests. Supplementary Table S3 contains SOPS distribution details.

#### Medication confound analyses

CPZeq were unrelated to ketamine similarity coefficients in ESZ (mean = 276.11 ± 341.88 mg; Spearman’s rho = −0.07, *p* = 0.57). CHR-P were not taking antipsychotic medication at the time of the study.

## Results

### Thalamic functional connectivity changes induced by ketamine

**Fig. 1:**
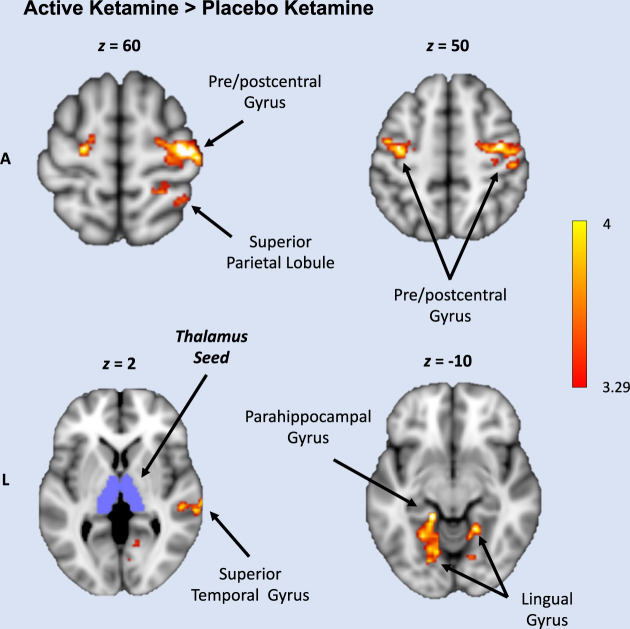
Connectivity increases following active ketamine infusion. Increased connectivity between bilateral thalamus (purple) and sensory regions for [active ketamine – saline] > [placebo ketamine – saline]. There were no significant clusters for [placebo ketamine – saline] < [ketamine ketamine – saline]. A anterior*,* L left. The color bar shows voxel-wise *z*-values. Coordinates (*x*, *y*, *z*) are reported in MNI space. Map is thresholded using a voxel-wise cluster defining threshold of *z* > 3.29 (*p* < 0.001) and FWE-corrected cluster significance threshold of *p* < 0.05.

Group-level maps illustrating regional connectivity with the thalamus during placebo ketamine and active ketamine are shown in Supplementary Fig. S3. Compared to placebo ketamine, active ketamine produced hyper-connectivity between the thalamus and seven non-contiguous clusters in motor, temporal, and occipital cortices (Fig. 1 and Supplementary Table S4). Active ketamine versus placebo ketamine comparisons for individual clusters are shown in Supplementary Fig. S4. Conversely, no regions showed greater thalamic connectivity on placebo ketamine relative to active ketamine.

### Effects of lamotrigine pre-treatment on ketamine-induced connectivity changes

**Fig. 2:**
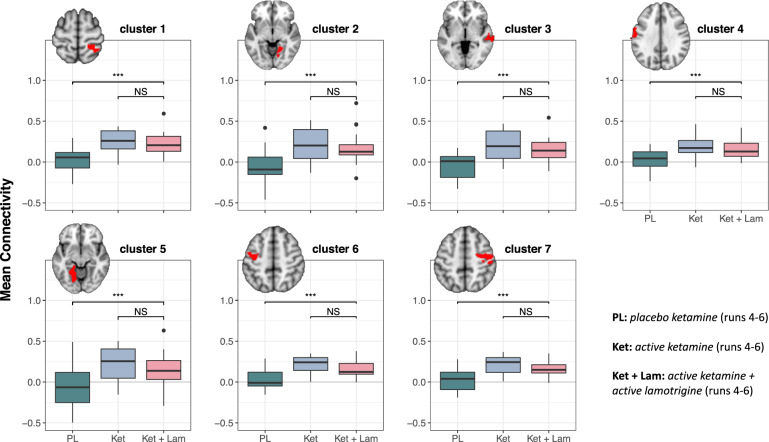
Distributions of placebo ketamine, active ketamine, and active lamotrigine + active ketamine connectivity from significant clusters. Boxplots showing distributions of participant-level connectivity means for each drug condition, for each significant cluster obtained from the [active ketamine – saline] > [placebo ketamine – saline] contrast (voxel-*z* > 3.29, corrected cluster-*p* < 0.05); anatomical location details for clusters 1 through 7 are found in Supplementary Table S4. PL placebo ketamine*,* Ket active ketamine, Lam active lamotrigine. Asterisks reflect significance levels from follow-up two-sided pairwise tests (based on the corresponding repeated measures ANOVA for that cluster). Uncorrected ***p < 0.001.

We assessed the effects of active lamotrigine versus placebo lamotrigine pre-treatment on ketamine-induced thalamic dysconnectivity within the seven regional clusters from the [active ketamine – saline] > [placebo ketamine – saline] contrast. Ketamine-induced hyper-connectivity appeared to be reduced with lamotrigine pre-treatment, but these differences were not significant (Fig. 2).

### Comparison of ketamine-induced versus schizophrenia thalamic dysconnectivity

**Fig. 3:**
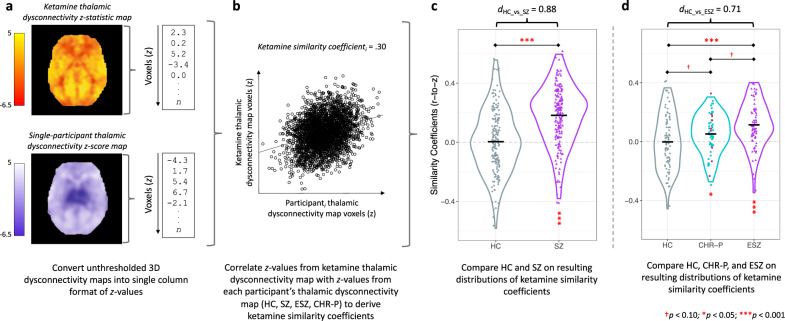
Visualization of the method used to correlate the ketamine-induced thalamic dysconnectivity pattern with thalamic connectivity in an independent data set of healthy control and schizophrenia participants. **a** The 3D group-level [active ketamine – saline] > [placebo ketamine – saline] thalamic dysconnectivity *z*-statistic map (orange) was converted into a 1D vector. Each healthy control and schizophrenia participant’s site- or age-corrected 3D thalamic dysconnectivity map (purple), in which voxel values were expressed as deviations from the normative values expected from the respective healthy control group, was converted into a 1D vector, with the same orientation as the ketamine-dysconnectivity map (to allow the two to be correlated). The color bars show voxel-wise *z*-values. **b** The [active ketamine – saline] > [placebo ketamine – saline] thalamic dysconnectivity *z*-statistic map was correlated with individual thalamic dysconnectivity maps to obtain a ketamine similarity coefficient for each healthy control and schizophrenia participant. **c** Ketamine similarity coefficients were significantly higher for individuals with schizophrenia relative to healthy controls; significance level (asterisks above the horizontal line) and Cohen’s *d* are based on an ANOVA comparing ketamine similarity coefficients across groups, controlling for sex and study site. Black horizontal lines represent within-group means, and vertical asterisks reflect significance levels for within-group tests comparing the mean similarity coefficient value against 0 (two-sided). **d** Ketamine similarity coefficients were significantly higher for early illness schizophrenia participants relative to healthy controls and those at clinical high-risk for psychosis; significance level (asterisks above the horizontal line) and Cohen’s *d* are based on an ANOVA model comparing ketamine similarity coefficients across groups, controlling for sex. Black horizontal lines represent within-group means, and vertical asterisks reflect significance levels for within-group tests comparing the mean similarity coefficient value against 0 (two-sided). Clinical high-risk for psychosis participants who converted to a psychotic disorder within 24 months of study entry (*n* = 10) are highlighted in maroon. HC healthy control participant, SZ schizophrenia participant, ESZ early illness schizophrenia participant, CHR-P clinical high-risk for psychosis participant.

The process of directly comparing the ketamine thalamic dysconnectivity map with rsfMRI thalamic connectivity maps from individual participants is illustrated in Fig. 3a, b.

As hypothesized, ketamine similarity coefficients were higher and more positive among SZ compared to HC (*t*_352_ = 8.34, *p* < 0.001, Cohen’s *d* = 0.88; Fig. 3c). Follow-up analyses revealed that similarity coefficients were, on average, greater than 0 for SZ (*t*_352_ = 10.48, *p* < 0.001), but did not differ from 0 for HC (*t*_352_ = −0.23, *p* = 0.82).

Ketamine similarity coefficients also differed across HC, CHR-P, and ESZ (*F*_2,200_ = 10.23, *p* < 0.001; Fig. 3d). ESZ had higher and more positive similarity coefficients compared to HC (*t*_200_ = 4.46, *p*_adj_ < 0.001; Cohen’s *d* = 0.71). CHR-P fell intermediately between, and showed trend-level differences from, ESZ (*t*_200_ = −1.93, *p*_adj_ = 0.06) and HC (*t*_200_ = 1.86, *p*_adj_ = 0.06). Similarity coefficients were, on average, greater than 0 for ESZ (*t*_200_ = 5.78, *p* < 0.001) and CHR-P (*t*_200_ = 2.13, *p* = 0.03), but not different from 0 for HC (*t*_200_ = −0.23, *p* = 0.82). Similarity coefficients did not significantly differ between CHR-P converters (*n* = 10) and non-converters followed for at least 24 months (*n* = 18); although 70% of converters had a similarity coefficient above the CHR-P group mean.

Ketamine similarity coefficients did not differ by sex in either clinical data set (Supplementary Materials; Supplementary Fig. S6).

For reference, ketamine similarity coefficient distributions for healthy males from the Ketamine – Lamotrigine Study are presented alongside the two clinical data sets in Supplementary Materials (Supplementary Fig. S7).

### Ketamine similarity coefficient correlations with clinical symptoms

**Fig. 4:**
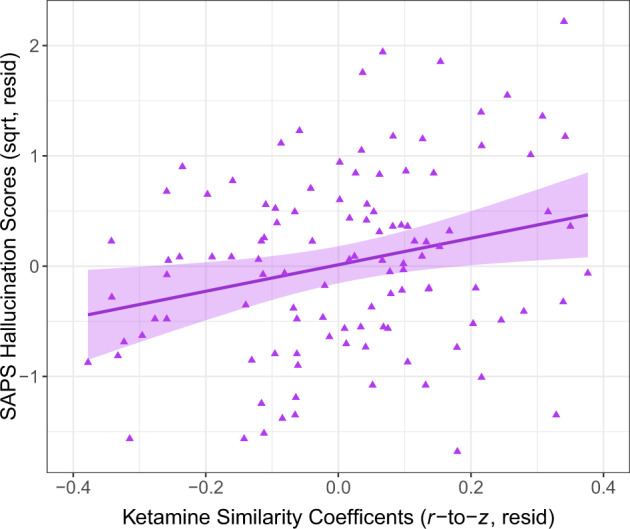
Relationships between ketamine similarity coefficients with clinical symptom ratings among schizophrenia participants. Relationship between SAPS Hallucination scores with ketamine similarity coefficients, controlling for the effects of sex and study site. SAPS Scale for the Assessment of Positive Symptoms, sqrt square-root, resid residualized.

Greater similarity to the ketamine-dysconnectivity pattern was associated with more severe Hallucination scores (*β*_105_ = 0.33, *p*_adj_ = 0.005; Fig. 4), a relationship that persisted when tested within the SZ subsample endorsing Hallucinations and taking antipsychotic medication (*β*_83_ = 0.27, *p* = 0.01), even after controlling for CPZeq (*β*_82_ = 0.28, *p* = 0.01). Conversely, similarity coefficients were unrelated to Delusions, Thought Disorder, Bizarre Behavior, or any negative symptoms (all *p*_adj_ > 0.70).

No significant sex differences in the relationship between ketamine similarity coefficients and symptom scores emerged (all *p*_adj_ > 0.10). Common slope tests between similarity coefficients and symptom scores were not significant for ESZ (all *p*_adj_ > 0.90) or CHR-P (all *p*_adj_ > 0.30).

## Discussion

Thalamic dysconnectivity is a robust neurobiological feature of SZ that is implicated in the development and presentation of psychotic disorders [7–9, 25, 26]. Administration of the NMDAR antagonist, ketamine, in healthy volunteers produced a thalamic dysconnectivity pattern resembling aspects of the thalamic dysconnectivity pattern seen in SZ [7]. Specifically, ketamine produced thalamic hyper-connectivity with several cortical sensory and motor regions similar to reports in SZ, but did not reproduce the thalamo-frontal and thalamo-cerebellar hypo-connectivities also observed in these reports [4, 5, 23, 24]. Ketamine-induced hyper-connectivity was not attenuated by pre-treatment with lamotrigine. Subsequently, and for the first time, we directly tested the relevance of the ketamine thalamic dysconnectivity model as a basis for thalamic dysconnectivity in SZ. The ketamine pattern was more like thalamic connectivity patterns seen in SZ than HC, with an intermediate relationship for CHR-P (relative to ESZ and a younger HC sample). Greater similarity to the ketamine pattern correlated with more severe hallucinations among more chronic SZ. Taken together, NMDAR antagonism via ketamine produces thalamic hyper-connectivity that resembles aspects of thalamic pathology seen in SZ and that appears to be clinically meaningful.

Thalamic connectivity changes induced by ketamine mirrored some, but not all, of the thalamic dysconnectivity patterns observed in SZ. That is, while thalamic connectivity to sensory and motor areas was enhanced by ketamine, connectivity with the prefrontal cortex and cerebellum was not reduced. This fits with notions that thalamic hyper-connectivity with sensory and motor regions is a particularly robust feature of SZ; for example, one study found that, when examining functional connections across the whole brain, hyper-connectivity of the thalamus with parietal, temporal, and visuospatial cortex was most discriminative of SZ [58]. Two recent studies similarly observed thalamic hyper- but not hypo-connectivity following ketamine administration [14, 16]. It is possible that only certain aspects of SZ thalamic dysfunction can be modeled via NMDAR blockade [16]. Alternatively, failures to detect thalamic hypo-connectivity with the prefrontal cortex and cerebellum (reported in SZ studies [6, 23, 24]) may be the result of acute rather than chronic ketamine dosing protocols. Ethical constraints prevent sub-chronic ketamine dosing in human experimental studies, limiting our understanding of chronic ketamine effects to naturalistic studies [31]. Chronic ketamine abuse can induce a clinical syndrome that is more severe, and more SZ-like, than the transient syndrome induced by acute doses [59]. For instance, chronic ketamine abusers exhibited reduced connectivity between the thalamus and prefrontal cortex relative to drug-free controls [60]; interestingly, this study also found thalamic hypo-connectivity with motor and parietal cortices, contrasting the hyper-connectivity induced by acute ketamine administration. Thus, longer-term NMDAR antagonism may drive thalamic hypo-, but not hyper-, connectivity.

In our study, ketamine-induced hyper-connectivity was not significantly reduced with lamotrigine pre-treatment. Lamotrigine is a sodium channel blocker that decreases glutamate release [61], and has been used to assess whether ketamine’s effects are mediated by downstream hyperglutmatergia [44]. Earlier studies found that lamotrigine pre-treatment attenuated ketamine-induced changes in the blood oxygenation level-dependent signal [62, 63] and some measures of functional connectivity [64] (but not others [17]). Our results could indicate that ketamine-induced thalamic hyper-connectivity is directly due to NMDAR antagonism as opposed to a more “downstream” increase in glutamate release [35]. That is, NMDAR antagonism by ketamine may increase the synchrony of neuronal firing across different brain regions but not necessarily increase the excitation level of the neuronal assemblies in those brain regions. Alternatively, ketamine may exert its effects by reducing the activation of somatostatin interneurons, which in turn, disinhibit fast-spiking parvalbumin GABAergic interneurons and increase resting gamma oscillations [32]; the resulting hyper-connectivity from parvalbumin neuro-dependent disinhibition is not driven by increased glutamate release, and consequently, would not be blocked by lamotrigine. Another possibility is that the lamotrigine dose was too small to overcome ketamine’s effects; while we did not have lamotrigine blood levels available to correlate with rsfMRI data, the failure to detect any significant effect of active lamotrigine versus placebo lamotrigine during saline infusion is consistent with an insufficient lamotrigine dose (see Supplementary Materials). Future research is needed to clarify how glutamate release contributes to ketamine-induced thalamic dysconnectivity.

A novel feature of our study is that we used ketamine-induced thalamic dysconnectivity to directly model thalamic dysconnectivity in SZ. When applied to an independent data set, ketamine thalamic dysconnectivity was more similar to thalamic connectivity exhibited by SZ than HC, a comparison yielding a relatively large effect size. A slightly smaller effect was observed in a separate sample of ESZ and HC, suggesting that similarity to the ketamine thalamic dysconnectivity pattern is present early in the SZ course and does not increase with illness chronicity. Nor is it accounted for by antipsychotic medication status or dose in these SZ samples. Ketamine thalamic dysconnectivity was marginally more similar to the patterns exhibited by CHR-P than HC, suggesting that NMDAR hypofunction-mediated thalamic dysconnectivity may predate psychosis onset, at least in some CHR-P. While greater similarity to the ketamine pattern did not differentiate future CHR-P converters from non-converters, this comparison was too underpowered to yield a definitive conclusion. In any case, ESZ showed marginally greater similarity to the ketamine pattern than CHR-P, suggesting that this similarity may intensify following SZ onset, and/or that it may be more evident in CHR-P who later transition to SZ.

In terms of symptom correlations, the greater the similarity between ketamine thalamic dysconnectivity and SZ dysconnectivity, the greater the hallucination severity in chronic SZ. This relationship was not present in ESZ, which had approximately one quarter the power to detect equivalent magnitude correlations; null symptom correlations among ESZ and CHR-P could relate to the less chronic and younger natures of those samples.

The thalamus coordinates and regulates information transfer through the cortex [65]. Excess connectivity could reflect an overabundance of incoming sensory information and/or a failure to regulate sensory information [66], giving rise to perceptual disturbances, like hallucinations. Our results add to previous findings from this sample that linked thalamus and temporal gyrus hyper-connectivity with greater hallucinatory behavior [23], and point to NMDAR hypofunction as a possible explanatory mechanism. Overlap with ketamine-induced thalamic dysconnectivity (principally sensory and motor hyper-connectivity) was unrelated to negative symptom severity. This is somewhat inconsistent with prior SZ studies that linked thalamo-sensory and thalamo-motor hyper-connectivity to negative symptoms [4, 67]. However, we correlated negative symptoms with ketamine similarity coefficients, producing one coefficient value per participant; this is fundamentally different from the voxel-wise correlation approach conducted in prior studies [4, 67]. Moreover, the acute negative symptoms induced by ketamine may only be superficially similar to the chronic negative symptoms associated with SZ [28].

### Limitations

First, the sample size for the ketamine study was relatively small, although the within-participant design provided sufficient power to detect significant effects. Second, to avoid risks of administering drugs during pregnancy, and to minimize influences of hormonal variation, females were excluded from the ketamine study; this limits the generalizability of our results. Third, while the ketamine sample was exclusively male, our clinical data sets included males and females. While larger ketamine similarity coefficients might be expected for males relative to females based on this, no such sex effects were observed: in both clinical data sets, and irrespective of diagnostic group, males and females did not significantly differ in their similarity coefficients (Supplementary Materials). Fourth, our ketamine template was derived from task-regressed data (versus the continuous rsfMRI data in our clinical data sets). The validity of cross-correlating task-regressed and continuous rsfMRI is supported by studies showing at least moderate correlations between these data types that are not much smaller than the test-retest reliabilities of true rsfMRI connectivity maps with themselves [68]. Moreover, differences between the approaches equally impacted all participants, so group differences could not be spuriously driven by attenuated correspondence between the two types of rsfMRI data. Finally, we found a small, but significant correlation between ketamine similarity coefficients and antipsychotic medication dosage in chronic SZ; thus, we cannot dismiss the possibility that medication influenced our between-group comparisons. However, we observed comparable group differences when comparing the early illness sample with less medication exposure for whom antipsychotic dosage was unrelated to similarity coefficients (and observed trend-level differences for CHR-P who were not taking medication).

## Conclusions

Using ketamine as a pharmacological probe, the current study presents compelling qualitative and quantitative evidence for a model of thalamic dysfunction in SZ arising from deficits in NMDAR signaling. These findings inform our understanding of the neurobiology, clinical features, and treatment of SZ. More broadly, this study illustrates the scientific value of using pharmacological probes to generate rsfMRI maps of specific neurotransmitter or receptor effects that in turn, can be used to investigate the pathophysiology of psychiatric conditions.

## Supplementary information

Supplementary Materials

## Data Availability

The unthresholded group-level ketamine template, i.e., [active ketamine – saline] > [placebo ketamine – saline] contrast map, is publicly available on NeuroVault (https://neurovault.org/collections/12212/).

## References

[1] Mitchell AS, Murray Sherman S, Sommer MA, Mair RG, Vertes RP, Chudasama Y (2014). Advances in understanding mechanisms of thalamic relays in cognition and behavior. J Neurosci.

[2] Halassa MM, Kastner S (2017). Thalamic functions in distributed cognitive control. Nat Neurosci.

[3] Anticevic A, Corlett PR, Cole MW, Savic A, Gancsos M, Tang Y (2015). N-methyl-D-aspartate receptor antagonist effects on prefrontal cortical connectivity better model early than chronic schizophrenia. Biol Psychiatry.

[4] Chen P, Ye E, Jin X, Zhu Y, Wang L. Association between thalamocortical functional connectivity abnormalities and cognitive deficits in schizophrenia. Sci Rep. 2019;9:2952. 10.1038/s41598-019-39367-z PMC6393449 30814558

[5] Woodward ND, Karbasforoushan H, Heckers S (2012). Thalamocortical dysconnectivity in schizophrenia. Am J Psychiatry.

[6] Woodward ND, Heckers S (2016). Mapping thalamocortical functional connectivity in chronic and early stages of psychotic disorders. Biol Psychiatry.

[7] Ramsay IS (2019). An activation likelihood estimate meta-analysis of thalamocortical dysconnectivity in psychosis. Biol Psychiatry Cogn Neurosci Neuroimaging.

[8] Giraldo-Chica M, Woodward ND (2017). Review of thalamocortical resting-state fMRI studies in schizophrenia. Schizophr Res.

[9] Pergola G, Selvaggi P, Trizio S, Bertolino A, Blasi G (2015). The role of the thalamus in schizophrenia from a neuroimaging perspective. Neurosci Biobehav Rev.

[10] Cronenwett WJ, Csernansky J. Thalamic pathology in schizophrenia. In: Swerdlow NR, editor. Behavioral neurobiology of schizophrenia and its treatment. Springer‐Verlag Berlin Heidelberg; 2010. p. 509–28.

[11] Clinton SM, Meador-Woodruff JH (2004). Thalamic dysfunction in schizophrenia: neurochemical, neuropathological, and in vivo imaging abnormalities. Schizophr Res.

[12] Krystal JH (1994). Subanesthetic effects of the noncompetitive NMDA antagonist, ketamine, in humans. Arch Gen Psychiatry.

[13] Abi-Saab WM, D’Souza DC, Moghaddam B, Krystal JH (1998). The NMDA antagonist model for schizophrenia: promise and pitfalls. Pharmacopsychiatry.

[14] Fleming LM, Javitt DC, Carter CS, Kantrowitz JT, Girgis RR, Kegeles LS, et al. A multicenter study of ketamine effects on functional connectivity: large scale network relationships, hubs and symptom mechanisms. NeuroImage Clin. 2019;22:101739. 10.1016/j.nicl.2019.101739 PMC6411494 30852397

[15] Driesen NR, McCarthy G, Bhagwagar Z, Bloch M, Calhoun V, D’Souza DC (2013). Relationship of resting brain hyperconnectivity and schizophrenia-like symptoms produced by the NMDA receptor antagonist ketamine in humans. Mol Psychiatry.

[16] Höflich A, Hahn A, Küblböck M, Kranz GS, Vanicek T, Windischberger C, et al. Ketamine-induced modulation of the thalamo-cortical network in healthy volunteers as a model for schizophrenia. Int J Neuropsychopharmacol. 2015;18:pyv040. 10.1093/ijnp/pyv040 PMC4576520 25896256

[17] Joules R, Doyle OM, Schwarz AJ, O’Daly OG, Brammer M, Williams SC (2015). Ketamine induces a robust whole-brain connectivity pattern that can be differentially modulated by drugs of different mechanism and clinical profile. Psychopharmacol (Berl).

[18] Barch DM (2014). Cerebellar-thalamic connectivity in schizophrenia. Schizophr Bull.

[19] Van Den Heuvel MP, Fornito A (2014). Brain networks in schizophrenia. Neuropsychol Rev.

[20] Anticevic A, Cole MW, Repovs G, Savic A, Driesen NR, Yang G, et al. Connectivity, pharmacology, and computation: toward a mechanistic understanding of neural system dysfunction in schizophrenia. Front Psychiatry. 2013;4:169. 10.3389/fpsyt.2013.00169 PMC3871997 24399974

[21] Cole MW, Pathak S, Schneider W (2010). Identifying the brain’s most globally connected regions. Neuroimage.

[22] Crossley NA, Mechelli A, Vértes PE, Winton-Brown TT, Patel AX, Ginestet CE (2013). Cognitive relevance of the community structure of the human brain functional coactivation network. Proc Natl Acad Sci USA.

[23] Ferri J, Ford JM, Roach BJ, Turner JA, Van Erp TG, Voyvodic J (2018). Resting-state thalamic dysconnectivity in schizophrenia and relationships with symptoms. Psychol Med.

[24] Anticevic A, Cole MW, Repovs G, Murray JD, Brumbaugh MS, Winkler AM (2014). Characterizing thalamo-cortical disturbances in Schizophrenia and bipolar illness. Cereb Cortex.

[25] Cao H, Chén OY, Chung Y, Forsyth JK, McEwen SC, Gee DG, et al. Cerebello-thalamo-cortical hyperconnectivity as a state-independent functional neural signature for psychosis prediction and characterization. Nat Commun. 2018;9:3836. 10.1038/s41467-018-06350-7 PMC6155100 30242220

[26] Anticevic A, Haut K, Murray JD, Repovs G, Yang GJ, Diehl C (2015). Association of thalamic dysconnectivity and conversion to psychosis in youth and young adults at elevated clinical risk. JAMA Psychiatry.

[27] Fryer S, Ferri J, Roach B, Loewy R, Stuart B, Anticevic A, et al. Thalamic dysconnectivity in the psychosis risk syndrome and early illness schizophrenia. Psychol Med. 2021:1–9. 10.1017/S0033291720004882. 10.1017/S0033291720004882 33719985

[28] Jentsch JD, Roth RH (1999). The neuropsychopharmacology of phencyclidine: from NMDA receptor hypofunction to the dopamine hypothesis of schizophrenia. Neuropsychopharmacology.

[29] Olney JW, Farber NB (1995). Glutamate receptor dysfunction and schizophrenia. Arch Gen Psychiatry.

[30] Javitt DC, Zukin SR, Heresco-Levy U, Umbricht D (2012). Has an angel shown the way? Etiological and therapeutic implications of the PCP/NMDA model of schizophrenia. Schizophr Bull.

[31] Gilmour G, Dix S, Fellini L, Gastambide F, Plath N, Steckler T (2012). NMDA receptors, cognition and schizophrenia – testing the validity of the NMDA receptor hypofunction hypothesis. Neuropharmacology.

[32] Krystal JH, Anticevic A, Yang GJ, Dragoi G, Driesen NR, Wang XJ (2017). Impaired tuning of neural ensembles and the pathophysiology of schizophrenia: a translational and computational neuroscience perspective. Biol Psychiatry.

[33] Cannon TD (2015). How schizophrenia develops: cognitive and brain mechanisms underlying onset of psychosis. Trends Cogn Sci.

[34] Keshavan MS, Anderson S, Pettergrew JW (1994). Is schizophrenia due to excessive synaptic pruning in the prefrontal cortex? The Feinberg hypothesis revisited. J Psychiatr Res.

[35] Moghaddam B, Adams B, Verma A, Daly D (1997). Activation of glutamatergic neurotransmission by ketamine: a novel step in the pathway from NMDA receptor blockade to dopaminergic and cognitive disruptions associated with the prefrontal cortex. J Neurosci.

[36] Schobel SA, Chaudhury NH, Khan UA, Paniagua B, Styner MA, Asllani I (2013). Imaging patients with psychosis and a mouse model establishes a spreading pattern of hippocampal dysfunction and implicates glutamate as a driver. Neuron.

[37] Javitt DC, Zukin SR (1991). Recent advances in the phencyclidine model of schizophrenia. Am J Psychiatry.

[38] Vukadinovic Z (2014). NMDA receptor hypofunction and the thalamus in schizophrenia. Physiol Behav.

[39] Kiss T, Hoffmann WE, Scott L, Kawabe TT, Milici AJ, Nilsen EA, et al. Role of thalamic projection in NMDA receptor-induced disruption of cortical slow oscillation and short-term plasticity. Front Psychiatry. 2011;2:14. 10.3389/fpsyt.2011.00014 PMC3089990 21556284

[40] Lisman J (2012). Excitation, inhibition, local oscillations, or large-scale loops: what causes the symptoms of schizophrenia?. Curr Opin Neurobiol.

[41] Wang X, Li Y, Chen J, Li Z, Li J, Qin L (2020). Aberrant auditory steady-state response of awake mice after single application of the NMDA receptor antagonist MK-801 into the medial geniculate body. Int J Neuropsychopharmacol.

[42] Thuné H, Recasens M, Uhlhaas PJ (2016). The 40-Hz auditory steady-state response in patients with schizophrenia a meta-analysis. JAMA Psychiatry.

[43] Leach MJ, Baxter MG, Critchley MAE (1991). Neurochemical and behavioral aspects of lamotrigine. Epilepsia.

[44] Anand A, Charney DS, Oren DA, Berman RM, Hu XS, Cappiello A (2000). Attenuation of the neuropsychiatric effects of ketamine with lamotrigine. Arch Gen Psychiatry.

[45] Chapman J, Chapman L, Kwapil T. Scales for the measurement of schizotypy. In: Raine A, Lencz TMS, editors. Schizotypal personality. New York: Cambridge University Press; 1995. p. 79–109.

[46] Anticevic A, Gancsos M, Murray JD, Repovs G, Driesen NR, Ennis DJ, et al. NMDA receptor function in large-scale anticorrelated neural systems with implications for cognition and schizophrenia. Proc Natl Acad Sci USA. 2012;109:16720–5. 10.1073/pnas.1208494109 PMC3478611 23012427

[47] Stone JM, Dietrich C, Edden R, Mehta MA, De Simoni S, Reed LJ (2012). Ketamine effects on brain GABA and glutamate levels with 1H-MRS: relationship to ketamine-induced psychopathology. Mol Psychiatry.

[48] Abdallah CG, De Feyter HM, Averill LA, Jiang L, Averill CL, Chowdhury GMI (2018). The effects of ketamine on prefrontal glutamate neurotransmission in healthy and depressed subjects. Neuropsychopharmacology.

[49] Elliott ML, Knodt AR, Cooke M, Kim MJ, Melzer TR, Keenan R (2019). General functional connectivity: shared features of resting-state and task fMRI drive reliable and heritable individual differences in functional brain networks. Neuroimage.

[50] Fair DA, Schlaggar BL, Cohen AL, Miezin FM, Dosenbach NUF, Wenger KK (2007). A method for using blocked and event-related fMRI data to study “resting state” functional connectivity. Neuroimage.

[51] Benjamini Y, Hochberg Y (1995). Controlling the false discovery rate: a practical and powerful approach to multiple testing. J R Stat Soc Ser B.

[52] Andreasen NC (1984). Scale for the assessment of positive symptoms (SAPS).

[53] Andreasen NC (1989). Scale for the assessment of negative symptoms (SANS).

[54] Heinks-Maldonado TH, Mathalon DH, Houde JF, Gray M, Faustman WO, Ford JM (2007). Relationship of imprecise corollary discharge in schizophrenia to auditory hallucinations. Arch Gen Psychiatry.

[55] Ford JM, Palzes VA, Roach BJ, Potkin SG, Van Erp TGM, Turner JA (2015). Visual hallucinations are associated with hyperconnectivity between the amygdala and visual cortex in people with a diagnosis of schizophrenia. Schizophr Bull.

[56] Woods SW (2003). Chlorpromazine equivalent doses for the newer atypical antipsychotics. J Clin Psychiatry.

[57] McGlashan T, Walsh B, Woods S (2010). The psychosis-risk syndrome: handbook for diagnosis and follow-up.

[58] Atluri G, Steinbach M, Lim KO, Kumar V, Macdonald A (2015). Connectivity cluster analysis for discovering discriminative subnetworks in schizophrenia. Hum Brain Mapp.

[59] Cheng WJ, Chen CH, Chen CK, Huang MC, Pietrzak RH, Krystal JH (2018). Similar psychotic and cognitive profile between ketamine dependence with persistent psychosis and schizophrenia. Schizophr Res.

[60] Liao Y, Tang J, Liu J, Xie A, Yang M, Johnson M (2016). Decreased thalamocortical connectivity in chronic ketamine users. PLoS One.

[61] Large CH, Webster EL, Goff DC (2005). The potential role of lamotrigine in schizophrenia. Psychopharmacol (Berl).

[62] Deakin JFW, Lees J, McKie S, Hallak JEC, Williams SR, Dursun SM (2008). Glutamate and the neural basis of the subjective effects of ketamine. Arch Gen Psychiatry.

[63] Doyle OM, De Simoni S, Schwarz AJ, Brittain C, O’Daly OG, Williams SCR, et al. Quantifying the attenuation of the ketamine pharmacological magnetic resonance imaging response in humans: a validation using antipsychotic and glutamatergic agents. J Pharmacol Exp Ther. 2013;345:151–60. 10.1124/jpet.112.201665 23370794

[64] Abdallah CG, Averill CL, Salas R, Averill LA, Baldwin PR, Krystal JH (2017). Prefrontal connectivity and glutamate transmission: relevance to depression pathophysiology and ketamine treatment. Biol Psychiatry Cogn Neurosci Neuroimaging.

[65] Saalmann YB, Pinsk MA, Wang L, Li X, Kastner S (2012). The pulvinar regulates information transmission between cortical areas based on attention demands. Science.

[66] Klingner CM, Langbein K, Dietzek M, Smesny S, Witte OW, Sauer H (2014). Thalamocortical connectivity during resting state in schizophrenia. Eur Arch Psychiatry Clin Neurosci.

[67] Cheng W, Palaniyappan L, Li M, Kendrick KM, Zhang J, Luo Q, et al. Voxel-based, brain-wide association study of aberrant functional connectivity in schizophrenia implicates thalamocortical circuitry. npj Schizophr. 2015;1:15016. 10.1038/npjschz.2015.16 PMC4849447 27336032

[68] Ganger S, Hahn A, Küblböck M, Kranz GS, Spies M, Vanicek T (2015). Comparison of continuously acquired resting state and extracted analogues from active tasks. Hum Brain Mapp.

