# Supplementary material for: Validation of ketamine as a pharmacological model of thalamic dysconnectivity across the illness course of schizophrenia
Source: Mol Psychiatry. 2022 Apr 14;27(5):2448–56. doi: 10.1038/s41380-022-01502-0 (PMC9135621; doi:10.1038/s41380-022-01502-0)
Supplement: Supplementary file 1 — Supplementary Materials [file 41380_2022_1502_MOESM1_ESM.pdf]

Supplemental Materials

Ketamine – Lamotrigine Study

Additional dosing information

Ketamine was supplied as ketamine hydrochloride 10mg/mL (Ketalar) in 10 mL vials from Amerisource, located in Springfield, Massachusetts, USA. Placebo to match ketamine was supplied as 0.9% sodium chloride (saline) solution, coded and bottled to be identical in appearance to active ketamine. Both ketamine and the saline placebo are colorless solutions that were placed in identically appearing syringes by the research pharmacist on each study day. The volumes of ketamine or saline placebo placed in each syringe were identical. Lamotrigine was supplied as a white to off white, flat faced, super-elliptical 100 mg chewable tablet. Placebo to match lamotrigine was identical to the lamotrigine 100 mg chewable tablet less the active ingredient.

Psychotomimetic effects of ketamine in healthy male volunteers

The Clinician-Administered Dissociative States Scale (CADSS[1]) and a selection of items from the Brief Psychiatric Rating Scale (BPRS[2]) were used to assess the psychotomimetic effects of ketamine in the sample of 18 healthy males. The CADSS and BPRS items were administered on each test day before the fMRI acquisition (baseline), and after the fMRI while the second infusion continued (see Table S1 for the study procedure timeline). Change scores relative to baseline were calculated for each test day. Change scores from the *Placebo Lamotrigine – Placebo Ketamine* test day were subtracted from change scores for each of the active ketamine test days. Using one sample *t*-tests, each active ketamine test day was then compared to 0. *Placebo Lamotrigine – Active Ketamine* yielded significant increases in total dissociative symptoms ($t_{17} = 6.79$, $p < .001$), as assessed with the CADSS, and significant increases in positive ($t_{17} = 3.19$, $p = .005$), negative ($t_{17} = 4.12$, $p < .001$), and disorganized symptoms ($t_{17} = 4.01$, $p < .001$), as assessed by the BPRS.

NMDAR antagonists, like ketamine, are believed to produce their psychotomimetic effects in humans by increasing glutamate release.[3] Accordingly, a prior study found that pre-treatment with lamotrigine, a glutamate release inhibitor, attenuated ketamine's neuropsychiatric and cognitive effects.[4] We therefore tested how pre-

treatment with oral lamotrigine impacted the psychotomimetic effects of ketamine in our data. Change scores calculated for the *Active Lamotrigine – Active Ketamine* test day similarly yielded increases in total dissociative symptoms (t_{17} = 5.45, p < .001), as well as positive (t_{17} = 4.37, p < .001), negative (t_{17} = 5.05, p < .001), and disorganized symptoms (t_{17} = 4.30, p < .001). However, when the two active ketamine test days were compared (i.e., with and without lamotrigine pre-treatment), no measures showed significant differences (all p > .10). Thus, while ketamine produced substantial increases in clinical/psychological symptoms, pre-treatment with lamotrigine did not significantly attenuate ketamine's effects.

Visual oddball task information

The visual oddball task presents a pseudorandom series of frequent standard stimuli (small blue circle against a white background; probability = .80), infrequent target stimuli (large blue circle against a white background; probability = .10), and novel stimuli (full-screen fractal patterns; probability = .10). All images were presented for 500 ms with fixed, 750 ms inter-stimulus intervals. Participants were instructed to press a response button to targets with their dominant hand index finger.

Neuroimaging data pre-processing and denoising

Neuroimaging data were pre-processed using FEAT in FSL version 6.0.0 (https://fsl.fmrib.ox.ac.uk/fsl/fslwiki/FEAT/). Initial steps included motion correction, slice-timing correction, and brain extraction. Next, the artifact detection toolbox (ART; http://www.nitrc.org/projects/artifact_detect/) and aCompCor[5] were used to denoise the functional magnetic resonance imaging (fMRI) data (in line with our earlier studies[6,7]). ART identifies fMRI volumes associated with outlier global intensity values (greater than z = 3) and excessive head motion (defined as translational motion greater than 2 mm in the x, y, or z plane or rotation motion greater than 0.02° in yaw, pitch, or roll). aCompCor uses principal components analysis (PCA) to derive components from the timeseries of voxels within the eroded white matter (WM) and cerebrospinal fluid (CSF). WM and CSF masks were derived from each participant's high-resolution T1-weighted structural images using Freesurfer (http://surfer.nmr.mgh.harvard.edu/) segmentation and then co-registered to their functional data; a union mask of WM and CSF noise voxels was created for each participant. A PCA was then applied to the timeseries for voxels within the noise mask to yield noise components that reflect weighted

averages of WM and CSF voxel timeseries. The subset of significant ($p < .05$) noise components, for each run, was selected using a Monte Carlo simulation[5] and retained for subsequent use as nuisance variables in the first-level connectivity analyses (described below). After implementing ART and aCompCor, FEAT was used to carry out high-pass temporal filtering (100 sec[8]) and spatial smoothing (6 mm kernel).

For each participant, the following nuisance regressors were entered in the first-level connectivity model: (i) seven motion parameters from ART (i.e., temporal derivatives of six motion parameters and the composite of total motion across translation and rotation), (ii) vectors flagging single outlier data points identified with ART to censor them, (iii) significant noise components from aCompCor, and (iv) task-condition event vectors convolved with the hemodynamic response function to regress out task-related activity.

Correlations between ketamine-induced thalamic dysconnectivity and ketamine-induced positive and negative symptoms in healthy male volunteers

As noted above, ketamine induced schizophrenia-like symptoms in a sample of healthy men. In separate group-level models using FSL's FEAT (https://fsl.fmrib.ox.ac.uk/fsl/fslwiki/FEAT/), we assessed whether ketamine-induced thalamic dysconnectivity effects correlated with ketamine-induced increases in positive or negative symptoms (assessed with the BPRS). For each participant, we created positive and negative symptom change scores that mimicked the between-day, within-day double contrasts from the main group-level analysis (e.g., positive symptoms during [active ketamine - saline] > [placebo ketamine - saline]); the resulting change scores were mean-centered. Subsequently, we regressed whole brain ketamine-induced thalamic dysconnectivity maps on ketamine-induced positive and negative symptom change scores in separate voxel-wise analyses. We used a voxel-wise height threshold of $z > 3.29$ ($p < .001$), and a more stringent corrected cluster significance threshold of $p < .025$, two-tailed, to account for the separate positive and negative symptom models (i.e., $p < .05/2$). These results were not significant, indicating that psychotomimetic effects in healthy volunteers were unrelated to thalamic connectivity changes.

Comparison of thalamus connectivity on saline and lamotrigine

Using region of interest (ROI) masks of significant regional clusters from the [placebo ketamine - saline] versus [active ketamine - saline] contrast, we compared thalamic connectivity from runs 1-3 across test days. Repeated measures ANOVAs were used to compare average thalamic connectivity in these ROIs across the three days; follow-up pairwise tests based on each ANOVA assessed (i) for differences in thalamic connectivity during saline infusion across test days where participants received placebo lamotrigine (i.e., *Placebo Lamotrigine – Placebo Ketamine* versus the *Placebo Lamotrigine – Active Ketamine* days), and (ii) whether active lamotrigine led to altered thalamic connectivity as compared to placebo lamotrigine (i.e., *Active Lamotrigine – Active Ketamine* versus the *Placebo Lamotrigine – Placebo Ketamine* and *Placebo Lamotrigine – Active Ketamine* days).

As shown in Figure S5, there were no differences in thalamic connectivity across test days during runs 1-3 (all ANOVAs, $p > .20$); this suggests that connectivity in these ROIs was reliable across test days and not impacted by the introduction of oral lamotrigine. The null lamotrigine finding is in line with an earlier report that used an equivalent, clinically effective lamotrigine dose (300 mg) and did not detect connectivity differences between lamotrigine and placebo.[9] Although this dose has been found to attenuate ketamine-induced changes in the blood oxygenation level-dependent (BOLD) signal[10] and global brain connectivity[11], it may not be sufficient for modulating ketamine's effects on thalamic connectivity, specifically. Alternatively, our study may be underpowered to detect these effects. Additional reasons for this null effect are discussed in the main text.

Ketamine similarity coefficient distributions in healthy male volunteers

We examined the strength and spread of the ketamine similarity coefficients by correlating the individual participant ketamine contrast maps with the group-level contrast map, i.e., the [active ketamine - saline] > [placebo ketamine - saline] contrast map. We also correlated the individual participant placebo ketamine map with the group-level contrast map, specifically runs 4-6 from the *Placebo Lamotrigine – Placebo Ketamine* test day; for this step, we first re-expressed voxel connectivity values for the individual placebo ketamine maps as z-scores, by subtracting the group mean and dividing by the group standard deviation. This procedure is

analogous to the healthy control participants (HC) z-score adjustments we used in the clinical data sets. The resulting distributions are shown in Figure S7a.

Further analyses indicated that ketamine similarity coefficients under placebo ketamine did not differ in variance from HC in the *Schizophrenia versus Healthy Control Study* data set ($F_{17,177}$ = 1.16, *p* = .60, Figure S7b), or HC in the *Early Illness Schizophrenia, Clinical High-Risk, versus Healthy Control Study* data set ($F_{17,84}$ = 1.55, *p* = .19; Figure S7c). Similarity coefficients for active ketamine did not differ in variance from clinical high-risk for psychosis participants (CHR-P) ($F_{17,44}$ = 1.03, *p* = .89), or early illness schizophrenia participants (ESZ) ($F_{17,73}$ = 0.78, *p* = .58); although there was a trend-level difference with schizophrenia participants (SZ) ($F_{17,182}$ = 0.45, *p* = .06).

These analyses show, as expected, that ketamine similarity coefficients were higher in the ketamine study participants (mean = 0.39, SD = 0.13) than in SZ (mean = 0.18, SD = 0.20) or ESZ (mean = 0.11, SD = 0.15), but that the variances were generally similar across the data sets.

We also note that the variance of similarity coefficients did not differ between HC and SZ in the *Schizophrenia versus Healthy Control Study* clinical data set ($F_{177,182}$ = 1.11, *p* = .48; Figure S7b). For ESZ and CHR-P from the *Early Illness Schizophrenia, Clinical High-risk, versus Healthy Control Study* clinical data set (Figure S7c), there was no significant difference in variance between HC and ESZ ($F_{84,73}$ = 1.44, *p* = .11), or ESZ and CHR-P ($F_{73,44}$ = 1.32, *p* = .32), but the CHR-P distribution was narrower than ESZ ($F_{84,44}$ = 1.90, *p* = .02).

Schizophrenia versus Healthy Control Study

Additional participant details

SZ met diagnostic criteria for schizophrenia based on the Structure Clinical Interview for DSM-IV-TR.[12] SZ and HC were excluded given a history of major medical illness, MRI contraindications, drug dependence in the past five years or current substance abuse, or an IQ less than 75. SZ with movement disorder symptoms were excluded (e.g., significant extra-pyramidal symptoms). Additionally, HC participants were excluded given a current or past history a of major neurological or psychiatric disorder, or a first-degree relative with a psychotic disorder diagnosis. The study protocol was approved by the Institutional Review Boards at the University of California, Irvine, the University of California, Los Angeles, the University of California, San Francisco, Duke University, the University of North Carolina, the University of New Mexico, the University of Iowa, and the University of Minnesota. Written informed consent was obtained from all participants; this included permission to share-de-identified data across study sites and with the broader research community.

Neuroimaging data denoising

As described in the *Ketamine – Lamotrigine Study*, ART and aCompCor were used to identify outlier volumes and noise components. Motion parameters from ART, vectors flagging outlier datapoints identified by ART, and significant noise components from aCompCor were entered as nuisance regressors in the first-level connectivity models.

Overlap between ketamine-induced thalamic dysconnectivity with schizophrenia thalamic dysconnectivity

In addition to the computation of ketamine similarity coefficients, we qualitatively evaluated the resemblance of the ketamine thalamic dysconnectivity pattern with the thalamic dysconnectivity pattern observed in schizophrenia. To this end, we performed a conjunction analysis of the group-level [active ketamine – saline] > [placebo ketamine – saline] contrast and the group-level SZ > HC contrast (previously reported in[6]). Both contrast maps were converted into z-maps, warped into MNI space, and thresholded (voxel-z > 3.29, corrected cluster-p < .05) using the same procedures to enable a direct comparison. Next, we identified which voxels passed this significance threshold for both contrasts.

Figure S2 shows the conjunction map, overlaid on the separate ketamine-induced thalamic dysconnectivity and schizophrenia thalamic dysconnectivity maps. We observed overlap in several sensory regions, including the superior temporal gyrus, pre and postcentral gyrus, lingual gyrus, and superior parietal lobule.

Ketamine similarity coefficients and sex

Because our ketamine template map was generated from only male participants, we tested for sex differences in the ketamine similarity coefficients derived from that template. Specifically, we used an ANOVA to test for sex differences in similarity coefficients between HC and SZ, while controlling for study site. There was no significant interaction between group and sex when predicting similarity coefficients ($F_{1,351} = 1.63$, $p = .20$), nor a main effect of sex after removing the non-significant interaction term ($F_{1,352} = 0.09$, $p = .77$). Figure S6 shows that the similarity coefficient means were similar across sexes for both groups.

Early Illness Schizophrenia, Clinical High-Risk, versus Healthy Control Study

Additional participant details

ESZ met diagnostic criteria for schizophrenia or schizoaffective disorder based on the Structure Clinical Interview for DSM-IV-TR.[12] ESZ were within 5 years of psychosis onset (mean = 1.72 ± 1.35 years). CHR-P met Criteria of Psychosis-risk Syndromes (COPS) based on the Structured Interview for Psychosis-Risk Syndromes (SIPS).[13–15] All participants were excluded for a DSM-IV past-year diagnosis of substance dependence (excluding nicotine), history of head injury with loss of consciousness, or a central nervous system disorder. HC were excluded if they met criteria for a major psychiatric disorder, or a first-degree relative with a psychotic disorder diagnosis. The study protocol was approved by the Institutional Review Board at the University of California, San Francisco. Written informed consent or assent was obtained from all participants.

Neuroimaging data denoising

ART and aCompCor were used to identify outlier volumes and noise components. Motion parameters from ART, vectors flagging outlier data points identified by ART, and significant noise components from aCompCor were entered as nuisance regressors in the first-level connectivity models.

Ketamine similarity coefficients and sex

We tested for sex differences in similarity coefficients across HC, CHR-P, and ESZ. As shown in Figure S6, there was no group X sex significant interaction ($F_{2,198} = 0.36$, $p = .70$), nor a main effect of sex after removing the non-significant interaction term ($F_{1,200} = 1.13$, $p = .29$).

References

1. Bremner JD, Krystal JH, Putnam FW, Southwick SM, Marmar C, Charney DS, et al. Measurement of dissociative states with the clinician-administered dissociative states scale (CADSS). J Trauma Stress. 1998;11:125–36.
2. Overall JE, Gorham DR. The Brief Psychiatric Rating Scale. Psychol Rep. 1962;10:799–812.
3. Moghaddam B, Adams B, Verma A, Daly D. Activation of glutamatergic neurotransmission by ketamine: A novel step in the pathway from NMDA receptor blockade to dopaminergic and cognitive disruptions associated with the prefrontal cortex. J Neurosci. 1997;17(8):2921–7.
4. Anand A, Charney DS, Oren DA, Berman RM, Hu XS, Cappiello A, et al. Attenuation of the Neuropsychiatric Effects of Ketamine With Lamotrigine. Arch Gen Psychiatry. 2000;57:270–6.
5. Behzadi Y, Restom K, Liau J, Liu TT. A component based noise correction method (CompCor) for BOLD and perfusion based fMRI. Neuroimage. 2007;37(1):90–101.
6. Ferri J, Ford JM, Roach BJ, Turner JA, Van Erp TG, Voyvodic J, et al. Resting-state thalamic dysconnectivity in schizophrenia and relationships with symptoms. Psychol Med. 2018;48(15):2492–9.
7. Abram S V., De Coster L, Roach BJ, Mueller BA, Van Erp TGM, Calhoun VD, et al. Oxytocin Enhances an Amygdala Circuit Associated with Negative Symptoms in Schizophrenia: A Single-Dose, Placebo-Controlled, Crossover, Randomized Control Trial. Schizophr Bull. 2020;46(3):661–9.
8. Poppe AB, Wisner K, Atluri G, Lim KO, Kumar V, Macdonald AW. Toward a neurometric foundation for probabilistic independent component analysis of fMRI data. Cogn Affect Behav Neurosci. 2013;13:641–59.
9. Joules R, Doyle OM, Schwarz AJ, O'Daly OG, Brammer M, Williams SC, et al. Ketamine induces a robust whole-brain connectivity pattern that can be differentially modulated by drugs of different mechanism and clinical profile. Psychopharmacology (Berl). 2015;232:4205–4218.
10. Doyle OM, De Simoni S, Schwarz AJ, Brittain C, O'Daly OG, Williams SCR, et al. Quantifying the attenuation of the ketamine pharmacological magnetic resonance imaging response in humans: A validation using antipsychotic and glutamatergic agents. J Pharmacol Exp Ther. 2013;345(151–160).
11. Abdallah CG, Averill CL, Salas R, Averill LA, Baldwin PR, Krystal JH, et al. Prefrontal Connectivity and Glutamate Transmission: Relevance to Depression Pathophysiology and Ketamine Treatment. Biol

Psychiatry Cogn Neurosci Neuroimaging [Internet]. 2017;2(7):566–74. Available from: https://doi.org/10.1016/j.bpsc.2017.04.006

12. First MB, Spitzer RL, Gibbon M, Williams JBW. Structured Clinical Interview for DSM-IV-TR Axis I Disorders, Research Version, Non-patient Edition. New York, NY: Biometrics Research, New York State Psychiatric Institute; 2002.

13. Miller TJ, McGlashan TH, Rosen JL, Somjee L, Markovich PJ, Stein K, et al. Prospective diagnosis of the initial prodrome for schizophrenia based on the structured interview for prodromal syndromes: Preliminary evidence of interrater reliability and predictive validity. Am J Psychiatry. 2002;159(5):863–5.

14. Miller TJ, McGlashan TH, Rosen JL, Cadenhead K, Ventura J, McFarlane W, et al. Prodromal Assessment with the Structured Interview for Prodromal Syndromes and the Scale of Prodromal Symptoms: Predictive Validity, Interrater Reliability, and Training to Reliability. Schizophr Bull. 2003;29(4):703–15.

15. McGlashan, T., Walsh, B., & Woods S. The psychosis-risk syndrome: Handbook for diagnosis and follow-up. New York, NY: Oxford University Press; 2010.

Table S1. Study Procedure Timeline

Time (minutes)	Procedure
-140	1st IV line placed
-90	Oral lamotrigine or placebo
-85	2nd IV line placed; infusion pump set up
-60	BPRS, CADSS
-40	Vital signs
-30	Participant placed in scanner
-20	Vital signs
-14	Acquire sagittal 3D MPRAGE
1	Saline bolus infusion plus infusion
8	Vital signs
10	Visual oddball runs 1 – 3
49	Ketamine or placebo bolus plus infusion
58	Visual oddball runs 4 – 6
89	Vital signs
90	Participant removed from scanner (infusion continues)
95	Vital signs
97	BRPS, CADSS
115	End of ketamine or placebo infusion; recovery period and discharge

Abbreviations: *BPRS*, Brief Psychiatric Rating Scale; *CADSS*, Clinician-Administered Dissociative States Scale.

Table S2. Clinical Symptom Subdomain Distributions in SZ and ESZ

SAPS/SANS Subdomain	SZ (*n*)[a]	Mean (SD)	ESZ (*n*)[a]	Mean (SD)
Hallucinations	114	12.43 ± 6.01	33	10.82 ± 5.28
Delusions	118	14.92 ± 8.26	37	13.42 ± 7.07
Thought Disorder	56	12.55 ± 5.79	17	9.24 ± 2.93
Bizarre Behavior	37	5.38 ± 2.18	8	6.00 ± 2.20
Affective Flattening	71	14.32 ± 6.22	43	15.67 ± 5.65
Alogia	51	7.96 ± 3.82	29	8.00 ± 3.26
Avolition/Apathy	116	9.13 ± 3.30	53	9.64 ± 2.85
Anhedonia/Asociality	124	12.25 ± 5.01	61	11.89 ± 5.04

[a]Participants with at least mild symptom severity (≥ 2) on the global rating from that subdomain (eliminating participants with symptoms rated absent or questionable).

Abbreviations: *SZ*, schizophrenia participant; *ESZ*, early illness schizophrenia participant.

Table S3. Clinical Symptom Subdomain Distributions in CHR-P

SOPS Subdomain	Mean (SD)
Positive	9.33 (4.17)
Negative	11.63 (5.94)
Disorganized	5.44 (3.38)
General	8.68 (4.56)

Abbreviations: *CHR-P*, clinical high-risk for psychosis participant.

Table S4. Significant Connectivity Clusters from Ketamine > Placebo Contrast

Cluster	Peak Voxel *z*	# Voxels	Anatomical Regions	MNI X	MNI Y	MNI Z
7	4.64	871	R Postcentral Gyrus	46	-16	58
			R Postcentral Gyrus	50	-20	60
			R Precentral Gyrus	46	-14	52
			R Precentral Gyrus	40	-10	50
			R Postcentral Gyrus	60	-14	42
			R Postcentral Gyrus	56	-14	44
6	4.56	456	L Precentral Gyrus	-30	-8	44
			L Precentral Gyrus	-22	-14	62
			L Precentral Gyrus	-36	-14	52
			L Precentral Gyrus	-46	-6	50
			L Superior Frontal Gyrus	-16	-6	66
			L Superior Frontal Gyrus	-18	-8	58
5	4.47	391	L Parahippocampal Gyrus, pos	-16	-38	-10
			L Lingual Gyrus	-8	-58	-2
			L Temporal Occipital Fusiform Cortex	-24	-54	-12
			L Lingual Gyrus	-18	-44	-8
			L Lingual Gyrus	-12	-66	-12
			L Lingual Gyrus	-18	-64	-10
4	4.36	348	L Postcentral Gyrus	-62	-12	24
			L Postcentral Gyrus	-56	-10	34
			L Precentral Gyrus	-60	-6	34
			L Postcentral Gyrus	-62	-14	30
			L Precentral Gyrus	-60	2	34
			L Parietal Operculum Cortex	-52	-26	24
3	4.53	318	R Middle Temporal Gyrus, pos	72	-24	-2

			R Superior Temporal Gyrus, pos	72	-24	4
			R Superior Temporal Gyrus, pos	68	-28	0
			R Middle Temporal Gyrus, pos	66	-32	0
			R Superior Temporal Gyrus, pos	54	-28	0
			R Superior Temporal Gyrus, pos	58	-20	-2
2	4.64	267	R Lingual Gyrus	20	-54	-4
			R Lingual Gyrus	14	-52	-12
			R Lingual Gyrus	10	-78	-2
			R Occipital Fusiform Gyrus	18	-72	-12
			R Lingual Gyrus	14	-70	-6
			R Lingual Gyrus	14	-60	2
1	4.47	231	R Superior Parietal Lobule	26	-46	68
			R Superior Parietal Lobule	32	-50	64
			R Postcentral Gyrus	34	-32	70
			R Superior Parietal Lobule	42	-46	60

Abbreviations: *L*, left; *R*, right; *pos*, posterior

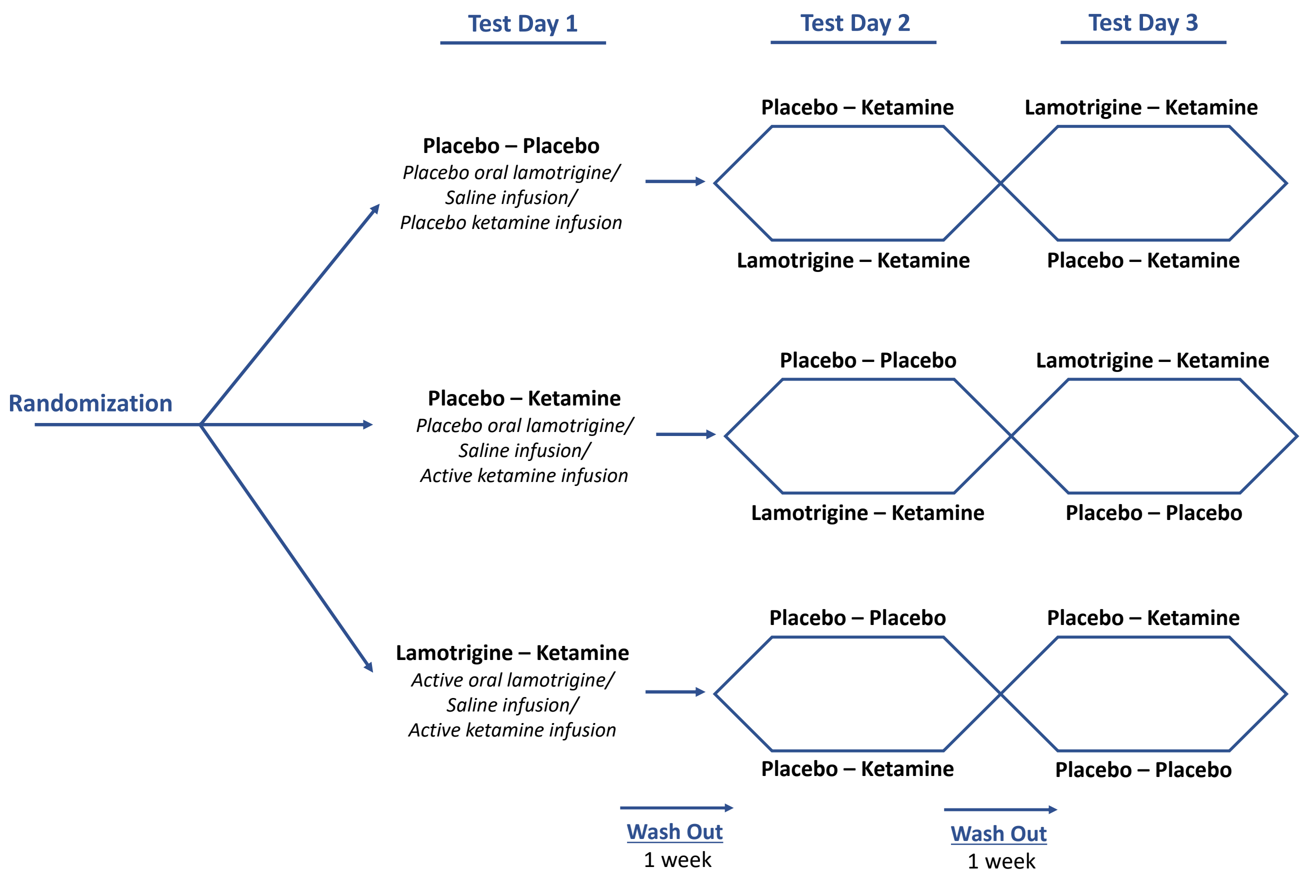

Figure S1. Visualization of randomized, double-blind, placebo-controlled, cross-over design. Participants were randomized to one of six drug orders. On each test day, participants were initially administered oral lamotrigine or oral placebo. Subsequently, participants were scanned twice: first during a single-blind intravenous placebo ketamine (saline) infusion, and second during a double-blind intravenous active ketamine or placebo ketamine (saline) infusion. There was at least one week in between treatments.

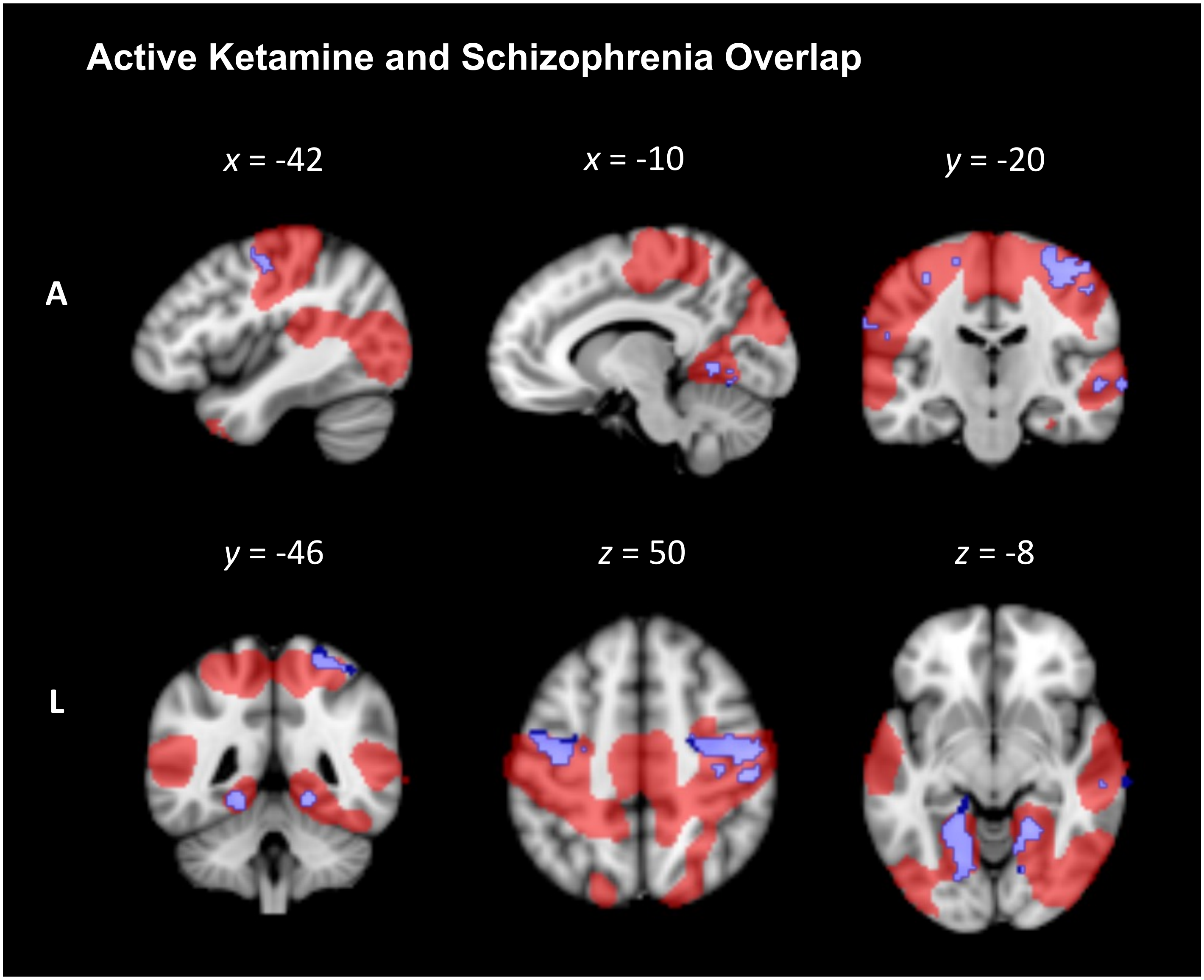

Figure S2. Overlap of ketamine-induced thalamic dysconnectivity and schizophrenia-related thalamic dysconnectivity. The [active ketamine - saline] > [placebo ketamine – saline] contrast map (*dark blue*) and the schizophrenia > healthy control contrast map (*red*) were each thresholded using a voxel-wise cluster defining threshold of $z > 3.29$ ($p < .001$) and FWE-corrected cluster significance threshold of $p < .05$. The conjunction of these maps (i.e., overlapping voxels) is shown in *light purple*. Coordinates are reported in MNI space.

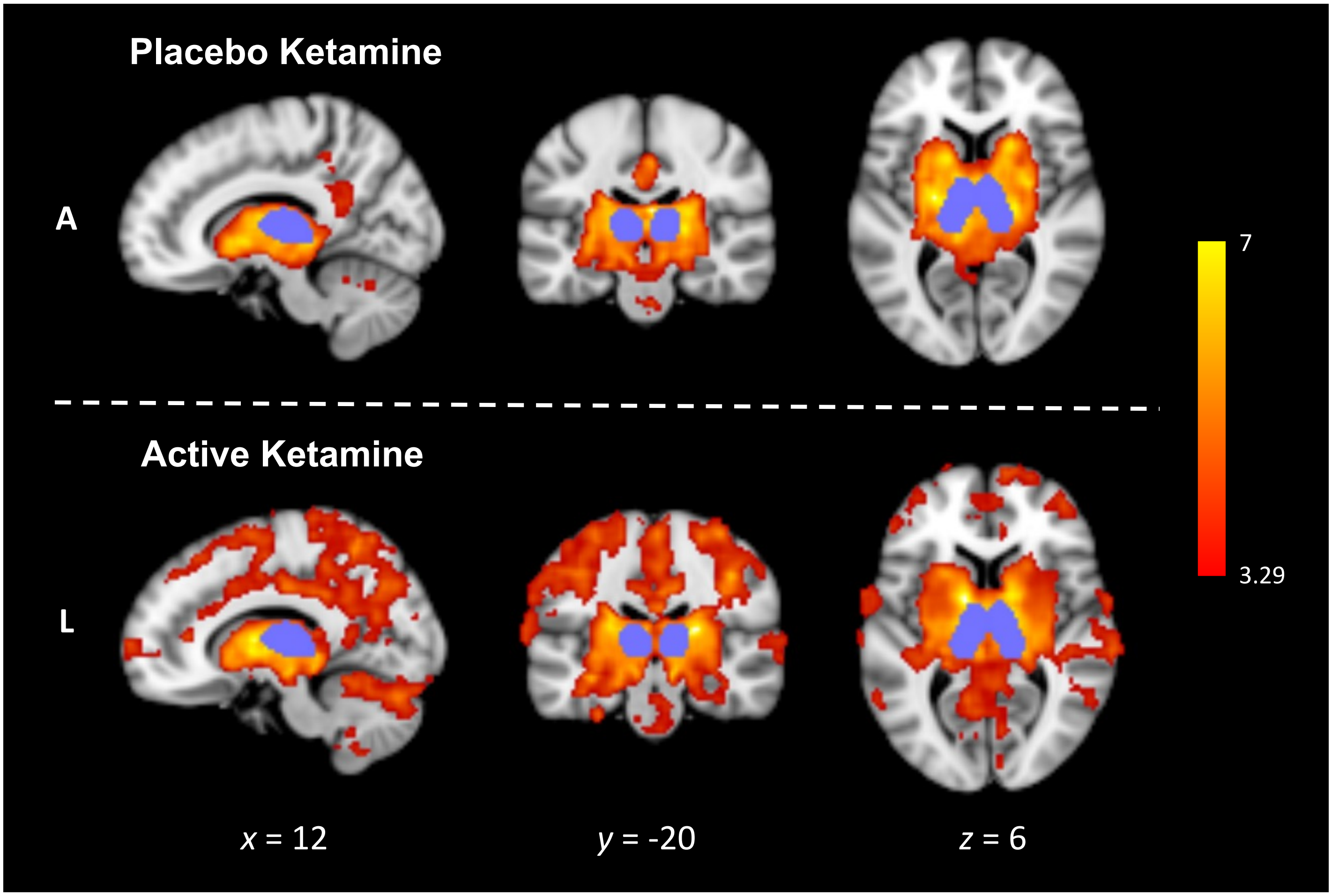

Figure S3. Regional connectivity with thalamus during placebo ketamine and active ketamine using a bilateral thalamus seed (*purple*). The color bar shows voxel-wise *z*-values. Coordinates are reported in MNI space. Map is thresholded using a voxel-wise cluster defining threshold of $z > 3.29$ ($p < .001$) and FWE-corrected cluster significance threshold of $p < .05$.

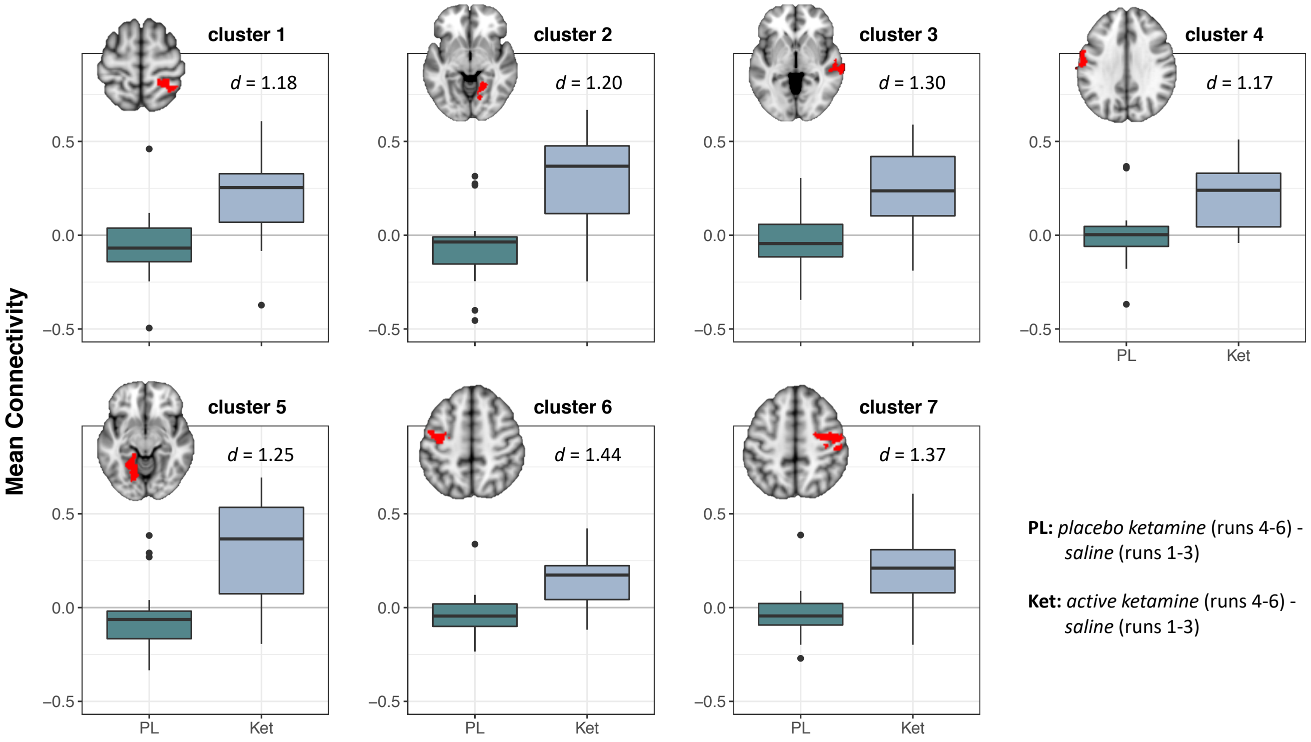

Figure S4. Distributions of placebo ketamine and active ketamine connectivity from significant clusters. Abbreviations: *PL,* placebo ketamine*; Ket,* active ketamine*.* Boxplots showing distributions of participant-level connectivity means from each within-day contrast, for each significant cluster obtained from the between-day [active ketamine - saline] > [placebo ketamine - saline] contrast (voxel-z > 3.29, corrected cluster-*p* < .05); anatomical location details for clusters 1 through 7 are found in Table S4. Cohen's *d* effect sizes are based on paired two-sided *t*-tests.

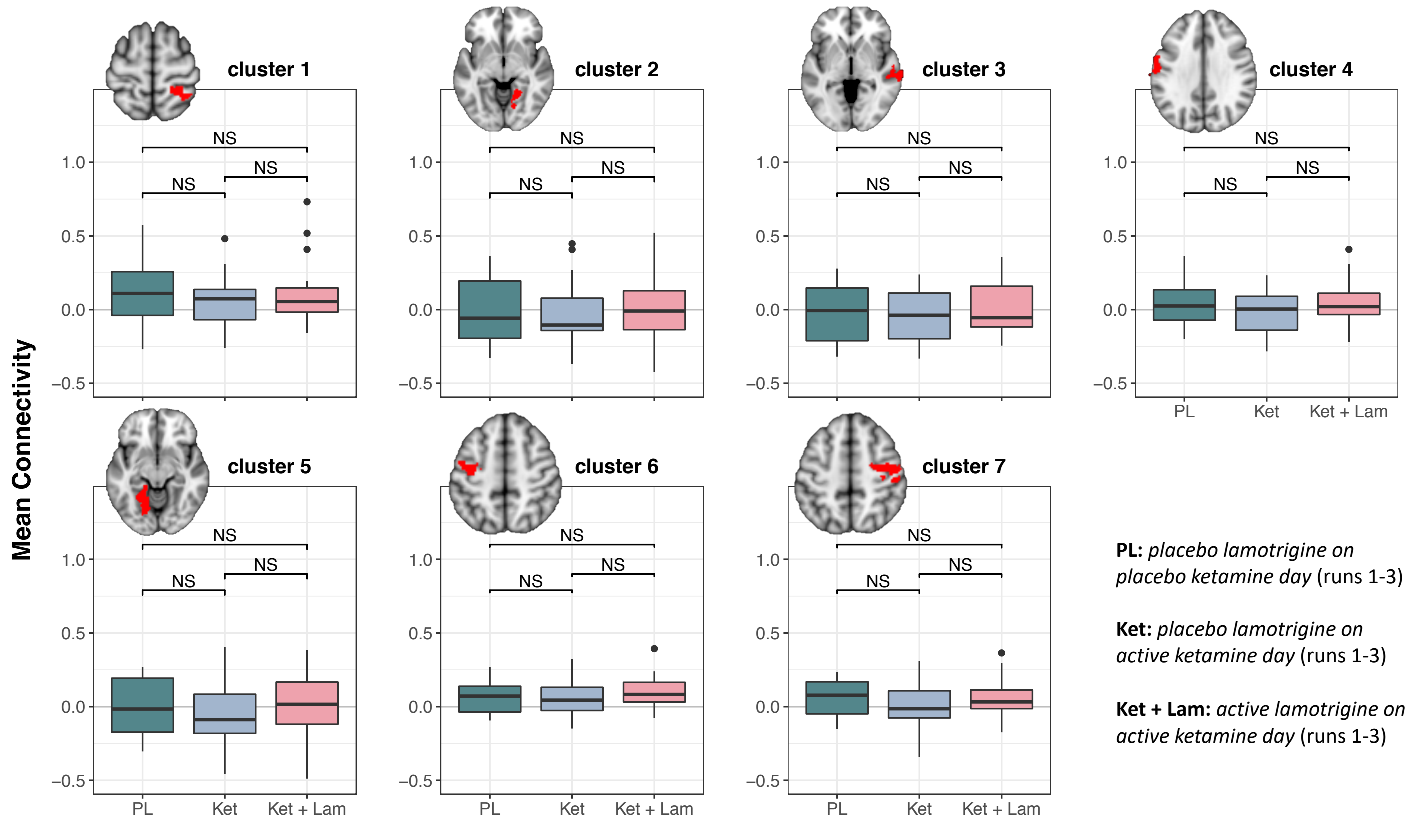

Figure S5. Distributions of placebo ketamine, active ketamine**,** and active lamotrigine + active ketamine connectivity from significant clusters. Abbreviations: *PL,* placebo ketamine*; Ket,* active ketamine; *Lam*, active lamotrigine. Boxplots showing distributions of participant-level connectivity means for runs 1-3 from each test day, for each significant cluster obtained from the [active ketamine - saline] > [placebo ketamine - saline] contrast (voxel-z > 3.29, corrected cluster-*p* < .05); anatomical location details for clusters 1 through 7 are found in Table S4. Asterisks reflect significance levels from follow-up pairwise tests (based on the corresponding repeated measures ANOVA for that cluster).

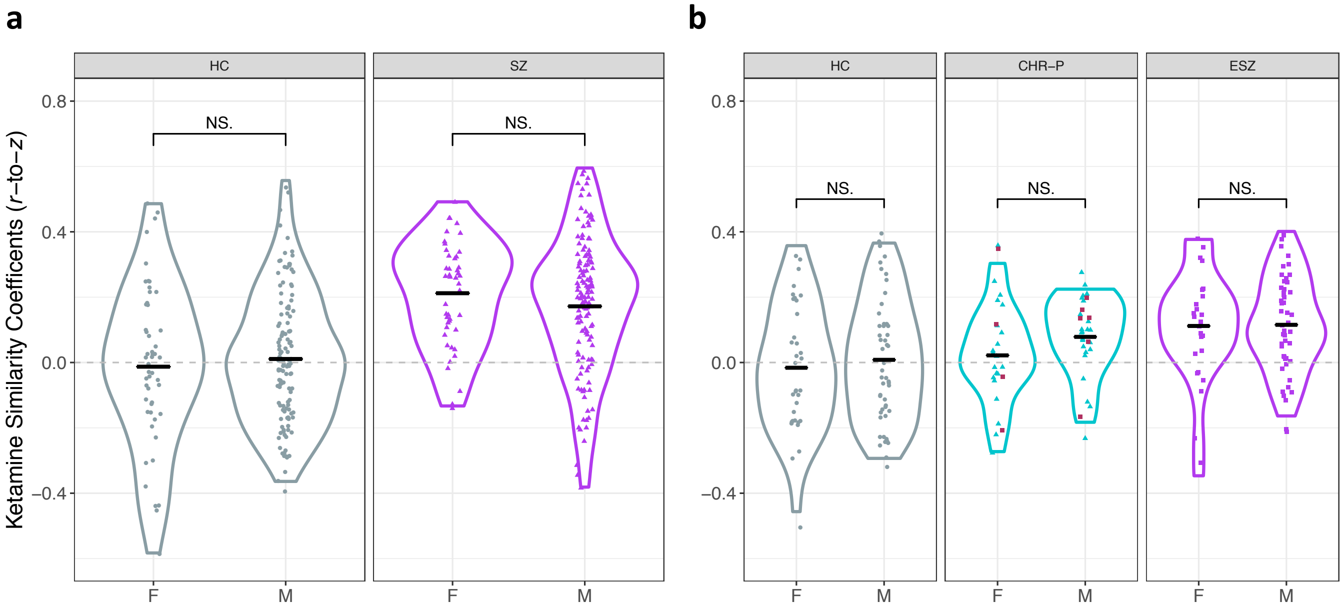

Figure S6. Sex distributions for ketamine similarity coefficients in each clinical data set. Abbreviations: *HC,* healthy control participant; *SZ,* schizophrenia participant; CHR-P; clinical high-risk for psychosis participant; *ESZ*, early schizophrenia participant*; M*, male; *F*, female. There were no sex differences, irrespective of diagnostic group, in either the **(a)** *Schizophrenia versus Healthy Control Study* data set, or the **(b)** *Early Illness Schizophrenia, Clinical High-Risk, versus Healthy Control Study* data set. CHR-P who converted to a psychotic disorder within 24 months of study entry are highlighted in maroon.

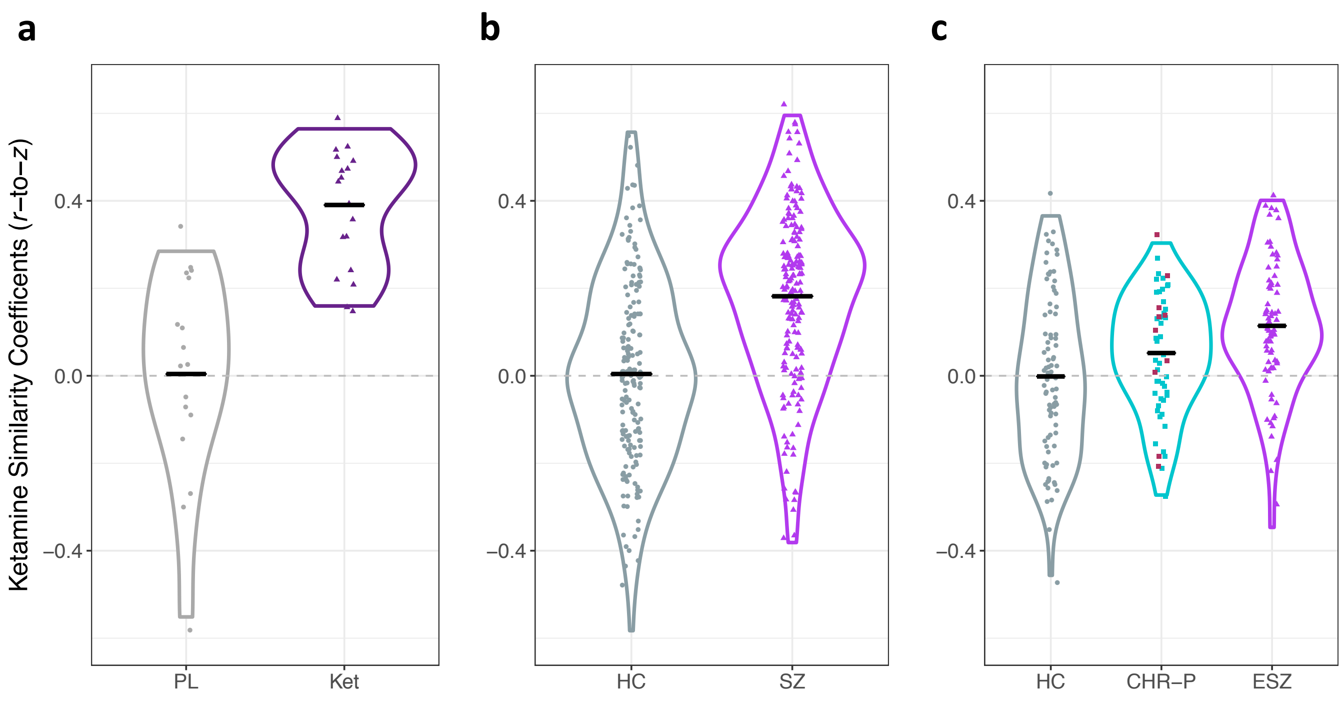

Figure S7. Ketamine similarity coefficient distributions in healthy male volunteers, compared to each clinical data set. Abbreviations: *PL,* placebo ketamine*; Ket,* active ketamine*; HC,* healthy control participant; *SZ,* schizophrenia participant; CHR-P; clinical high-risk for psychosis participant; *ESZ*, early illness schizophrenia participant. **(a)** Similarity coefficient distributions under placebo ketamine and active ketamine (i.e., [active ketamine - saline] > [placebo ketamine - saline]). **(b, c)** Distributions from the two clinical data sets are provided here for reference. CHR-P who converted to a psychotic disorder within 24 months of study entry are highlighted in maroon **(c)**.
